# Molecular and phenotypic characterization of seedling and adult plant leaf rust resistance in a world wheat collection

**DOI:** 10.1007/s11032-013-9899-8

**Published:** 2013-08-11

**Authors:** Abdulsalam Dakouri, Brent D. McCallum, Natasa Radovanovic, Sylvie Cloutier

**Affiliations:** 1 Cereal Research Centre, Agriculture and Agri-Food Canada, 195 Dafoe Road, Winnipeg, MB R3T 2M9 Canada; 2 Department of Plant Science, University of Manitoba, 66 Dafoe Road, Winnipeg, MB R3T 2N2 Canada

**Keywords:** *Triticum aestivum*, *Puccinia triticina*, *Lr* genes, Gene postulation, APR genes

## Abstract

Genetic resistance is the most effective approach to managing wheat leaf rust. The aim of this study was to characterize seedling and adult plant leaf rust resistance of a world wheat collection. Using controlled inoculation with ten races of *Puccinia triticina*, 14 seedling resistance genes were determined or postulated to be present in the collection. *Lr1*, *Lr3*, *Lr10* and *Lr20* were the most prevalent genes around the world while *Lr9*, *Lr14b*, *Lr3ka* and/or *Lr30* and *Lr26* were rare. To confirm some gene postulations, the collection was screened with gene-specific molecular markers for *Lr1*, *Lr10*, *Lr21* and *Lr34*. Although possessing the *Lr1* and/or *Lr10* gene-specific marker, 51 accessions showed unexpected high infection types to *P. triticina* race BBBD. The collection was tested in the field, where rust resistance ranged from nearly immune or highly resistant with severity of 1 % and resistant host response to highly susceptible with severity of 84 % and susceptible host response. The majority of the accessions possessing the adult plant resistance (APR) gene *Lr34* had a maximum rust severity of 0–35 %, similar to or better than accession RL6058, a Thatcher-*Lr34* near-isogenic line. Many accessions displayed an immune response or a high level of resistance under field conditions, likely as a result of synergy between APR genes or between APR and seedling resistance genes. However, accessions with three or more seedling resistance genes had an overall lower field severity than those with two or fewer. Immune or highly resistant accessions are potential sources for improvement of leaf rust resistance. In addition, some lines were postulated to have known but unidentified genes/alleles or novel genes, also constituting potentially important sources of novel resistance.

**Electronic supplementary material:**

The online version of this article (doi:10.1007/s11032-013-9899-8) contains supplementary material, which is available to authorized users.

## Introduction

Leaf rust, caused by the fungal pathogen *Puccinia triticina* Eriks., has been the most prevalent disease in wheat-producing areas (Kolmer 2005). This fungus is adapted to a wide range of environments and it can co-exist with wheat wherever it is grown (Winzeler et al. 2000). It can cause significant yield loss, reaching 15 % or more depending on the crop developmental stage at infection and the susceptibility of the cultivars (Samborski 1985). Genetic resistance has been the most effective method of controlling this disease because it constitutes an environmentally friendly and cost-effective long-term strategy for minimizing yield losses (Pink 2002).

Leaf rust resistance genes named *Lr1* to *Lr68* have been characterized in bread wheat, durum wheat and diploid wheat species. These genes are located on 20 of the 21 chromosomes of hexaploid wheat (McIntosh et al. 1995, 2007; McCallum et al. 2012). Most *Lr* genes confer race-specific resistance and follow the gene-for-gene concept leading to a hypersensitive response (HR) or programmed cell death (Flor 1942). Through co-evolution of host and pathogen, most of these genes have been overcome by new pathogen races. Between 1938 and 1964, Australia released many varieties containing single *Lr* genes, which consequently resulted in an increase in the frequency of corresponding virulent isolates of *P. triticina* (Park et al. 2001). Gene pyramiding is therefore a viable strategy to provide more durable resistance by slowing down the rate at which single resistance genes are overcome.

*Lr* seedling resistance genes may be postulated upon inoculation with leaf rust isolates with known avirulence and virulence patterns on wheat differential sets, i.e., cultivars with known *Lr* genes. Initially established by Loegering et al. (1971) and Browder (1973), gene postulation has been a method of choice for several decades. This method was used to survey leaf rust resistance genes in several collections including a world collection of winter wheat (McVey 1992), American hard red spring wheat (Statler 1984; Oelke and Kolmer 2004), American hard red winter wheat (McVey and Long 1993), American soft red spring wheat (Kolmer 2003; Wamishe and Milus 2004), Mexican wheat cultivars (Singh 1993b; Singh and Rajaram 1991), cultivars from Ethiopia and Germany (Mebrate et al. 2008), Chinese cultivars (Li et al. 2010; Singh et al. 1999), British cultivars (Singh et al. 2001), Argentinean wheat cultivars (Vanzetti et al. 2011) and Eastern, Western and Northern European germplasm (Bartos and Valkoun 1988; Herrera Foessel 2001; Park et al. 2001; Winzeler et al. 2000).

A few *Lr* genes conferring resistance at the adult plant stage have also been characterized. These genes include *Lr12* (McIntosh and Baker 1966), *Lr13* (Dyck et al. 1966), *Lr22a* and *b* (Dyck 1979), *Lr34* (Dyck 1987), *Lr35* (Kerber and Dyck 1990), *Lr37* (Bariana and McIntosh 1993), *Lr46* (Singh et al. 1998), *Lr67* (Herrera-Foessel et al. 2011; Hiebert et al. 2010), *Lr68* (Herrera-Foessel et al. 2012) and more recently *trp*-*1* and *trp*-*2* (Da-Silva et al. 2012). Genes *Lr34*, *Lr46* and *Lr67* provide partial or slow resistance to leaf rust and are considered more durable than seedling resistance genes (Caldwell 1968). The mode of action of these genes is characterized by a longer latent period, a lower infection frequency, smaller uredinia size, a shorter period of sporulation and a lower spore density (Caldwell 1968). Among these genes, *Lr34* has not only been durable but it has also been demonstrated to act synergistically with other leaf rust resistance genes (German and Kolmer 1992) and has a pleiotropic effect on other diseases (Singh 1992b; Spielmeyer et al. 2005).

Efficient utilization of genetic resistance relies on an accurate and deep knowledge of the leaf rust resistance genes or gene combinations and of their effectiveness in different environments. This knowledge would undoubtedly raise our understanding of the durability of the genes and assist in pyramiding resistance genes in adapted germplasm. Only four *Lr* resistance genes have been cloned and sequenced to date: *Lr1* (Cloutier et al. 2007), *Lr10* (Feuillet et al. 2003), *Lr21* (Huang et al. 2003) and *Lr34* (Krattinger et al. 2009). Molecular markers have been developed for each of them based on their sequence (Schachermayr et al. 1997; Huang and Gill 2001; Cloutier et al. 2007; Lagudah et al. 2009; Dakouri et al. 2010). Molecular markers for other *Lr* genes have been developed but these would be linked and not necessarily perfectly diagnostic (for review see McCallum et al. 2012). The goals of the current study were to characterize the seedling and adult plant *Lr* resistance genes present in a world collection (WC), to evaluate their effectiveness under field conditions and to correlate the results with gene-specific molecular markers in order to validate the postulation and to also identify potential sources of novel resistance and superior gene combinations for use in breeding programs.

## Materials and methods

### Seedling resistance gene analysis

A total of 275 wheat accessions representing 42 countries were surveyed (Supplementary data Table S1). These lines include wheat cultivars, breeding lines and landraces of hexaploid wheat. Thirty Thatcher near-isogenic lines (NILs) of wheat with known *Lr* genes constituted the differential set (Table 1).

**Table 1:** Seedling infection types observed on the 30 Thatcher near-isogenic lines constituting the differential set upon inoculation with 10 races of *Puccinia triticina*

Differential sets	*Lr* gene	BBBD	MBDS	MGBJ	TJBJ	TDBG	MBRJ	PBDQ	THMJ	TNRJ	TCRJ
RL 6003	*Lr1*	0	3	3	3	3	33+	33+	3	3	3
RL 6016	*Lr2a*	0	0	0	3	33+	0	2	3	3	3
RL 6019	*Lr2b*	0	0	0	3	3	1	2	3	3	3
RL 6047	*Lr2c*	1	1	1	3++	3	0	3	3	3	3
RL 6002	*Lr3*	1−	3−	3	3	3	33+	3	3	3	3
RL 6010	*Lr9*	0	0	0	0	0	;	0	0	3	0
RL 6005	*Lr16*	1+	2+	3−	3	2	22+	;1	3	2	2
RL 6064	*Lr24*	0	0	0	3++	33−	0	0	;	3	;
RL 6078	*Lr26*	1−	2+	1	2	23	;	2	3	;	3
RL 6007	*Lr3Ka*	1	2	2	2	2	33+	2	3	3	3
RL 6053	*Lr11*	2	2+	2	2+	2	33+	2	2	3	3
RL 6008	*Lr17*	1	3	1	1+	21	2	3	;1	0	;1
RL 6049	*Lr30*	2	2	2	2+	2	33+	2	3	3	3
RL 6051	*LrB*	2	3	2+	2+	23	23	3	2	2	2
RL 6042	*Lr3bg*	;	3	3	2	2	2	3	X	X	X
RL 6004	*Lr10*	1−	3	3−	3	33−	33+	3	3	3	3
RL 6013	*Lr14a*	3	3	3	3	X	33+	2	3	3	3
RL 6006	*Lr14b*	3	3	3	3	X	33+	;1	3	0	3
RL 6052	*Lr15*	3−	3	3	3	33+	33+	3	3	2	3
RL 6009	*Lr18*	2	2+	1	2	21	22+	22+	22+	2	23
RL 6040	*Lr19*	0	0	0	0	0	0	0	0	0	0
RL 6092	*Lr20*	3	3	3	3	2	33+	3	3	3	3
RL 6043	*Lr21*	2	2+	1	1	2	;	2	2	0	2
RL 6012	*Lr23*	3−	3	2	3	33+	33+	2	3	0	3
RL 6094	*Lr25*	0	0	0	1	;	;	3	0	0	2
RL 6079	*Lr28*	0	0	3	3	33+	33+	33+	3	3	3
RL 6080	*Lr29*	1	1	1	1	13−	2	x	;2	;	2
RL 5497	*Lr32*	2	2	1	1+	2	3	x	x	0	2
RL 6107	*Lr52*	2	2	2+	1	33–	33+	;	;	0	;1
Thatcher	–	3	3	3	3++	33+	33+	3	3	3	3

Infection types: 0 = no flecks or uredinia, 0; = faint hypersensitive flecks, ; = hypersensitive flecks, 1 = small uredinia with necrosis, 2 = small to medium uredinia with necrosis, 3 = moderate to large size uredinia with/without chlorosis, 4 = very large uredinia without chlorosis, X = mesothetic, a mixture of resistant pustule types, “+” = indicates slightly larger uredinia, “−” = indicates slightly smaller uredinia, infection types (ITs) with two symbols denote a range: e.g., 22+ = indicates a mixture of 2 sizes of uredinia with chlorosis and slightly larger uredinia with chlorosis

The following ten races of the leaf rust pathogen *Puccinia triticina* were tested on the wheat germplasm and on the differential set: BBBD, MBDS, MGBJ, TJBJ, TDBG, MBRJ, PBDQ, THMJ, TNRJ and TCRJ. The infection type of these races onto the differentials is listed in Table 1.

Five seeds from each accession were planted in 30 × 25 cm fiber flats with 25 accessions in each flat. A mixture of soil, sand and peat moss was used as a growing medium. The fungicide Captan was applied at seeding to prevent fungal infection at germination. The seeded flats were placed in a greenhouse (20–25 °C and 16 h light) and watered as required. Twelve days after planting, i.e., at the two-leaf stage, the seedlings were inoculated with a single race. The inoculation was conducted by spraying the spore solution onto the seedlings using micro-inoculators (University of Minnesota, Minneapolis, USA). The inoculated seedlings were left at ambient environment for 1 h prior to being placed in a humidity chamber for 24 h under conditions of 100 % humidity and 20 °C. After incubation, the flats were returned to the greenhouse and watered daily. Fourteen days after inoculation (DAI), the infection types (ITs) were scored for each race using the 0–4 modified Stakman Scale (Roelfs and Singh 1992). ITs of 3 or higher refer to high infection types (HITs) while ITs of 2 or less were low infection types (LITs). The seedling resistance gene postulation was conducted by comparing the IT patterns of the WC accessions to those of the differential set. Five plants of each accession were scored for their ITs. Seedling tests for lines showing discrepancies between molecular markers and gene postulation were repeated twice for confirmation.

### Molecular markers

DNA extraction of the WC was performed as described by Dakouri et al. (2010). Molecular markers specific to *Lr1* (Cloutier et al. 2007), *Lr10* (Schachermayr et al. 1997), *Lr21* (Huang and Gill 2001) and *Lr34* (Dakouri et al. 2010) were assessed to confirm the presence or absence of these genes in the WC. Primer names, sequences, PCR conditions and references for each marker are listed (Supplementary data Table S2). For *Lr34*, the molecular markers caIND11, caSNP4 and caSNP12 were assessed as previously described (Dakouri et al. 2010). *Lr1*, *Lr10* and *Lr21* PCR reactions were performed in10 μl aliquots containing 60 ng genomic DNA, 1× PCR buffer, 0.8 mM dNTPs, 1.5 mM MgCl_2_, 0.4 μM each forward and reverse primers, 0.1 μl of 10× bovine serum albumin (1 mg/mL) and 1 U *Taq* DNA polymerase. Glenlea, near-isogenic line (NIL) RL6004 (Thatcher-*Lr10*), McKenzie and NIL RL6058 (Thatcher-*Lr34*) were used as positive controls for *Lr1*, *Lr10*, *Lr21* and *Lr34*, respectively. The PCR products for *Lr1*, *Lr10*, *Lr21* and *Lr34* markers caSNP4 and caSNP12 were resolved on 1.5 % agarose gel while *Lr34* marker caIND11 was resolved on an ABI3130xl (Applied Biosystems, Foster City, CA, USA) as previously described (Dakouri et al. 2010).

### Field experiments

The world collection was planted in a randomized complete block design (RCBD) with two replications at each of three locations, namely Winnipeg (WPG), Glenlea (GLN) and Portage La Prairie (POR), Manitoba, Canada in 2009, 2010 and 2011. Ten seeds of each accession were planted per hill in WPG and GLN and single 50-cm rows in POR with 25 cm between hills or rows. The wheat cultivars Mckenzie, Thatcher and Thatcher-*Lr34* NIL RL6058 were used as checks. Spreader rows of the leaf rust susceptible cultivar Morocco were planted between groups of five rows or hills. Leaf rust epidemics were established by inoculating the entire experiments with a mixture of leaf rust races. In 2009, a mixture of the following races was used: MBDS, MBPS, MBTS, MDNS, MDPS, MFDS, MFNS, MFPS, MLDS, PDBB, TDBG, TDBJ, TFBJ, TFBS, TGBJ, TJBG and TLDS. In 2010, the following races were used: MBPS, MDNS, MDPS, MFDS, MFNS, MFPS, MJDS, MLDS, MNDS, PBDG, PBDQ, TBBJ, TBJS, TDBG, TDBJ, TFBG, TMGJ, TNBQ and TNPS. In 2011, the following races were used: MBPS, MCNS, MDDS, MDNS, MDPS, MDTS, MFDS, MFNS, MFPS, MLDS, PBDQ, PBJQ, TBBG, TBBJ, TDBG, TDBJ, TDGJ, TNBG and TNBJ. Rust severity ratings and reaction types using a modified Cobb scale (Peterson et al. 1948) were collected at maximum rust severity (MRS), which was determined to be when the susceptible check Thatcher showed maximum severity. The host reaction type was evaluated as follows: R, no uredinia present; MR, small uredinia with necrosis and light sporulation; MR-MS, small to medium-size uredinia with moderate sporulation; MS, medium-size uredinia with moderate to intensive sporulation; S, large uredinia with abundant sporulation.

### Data analysis

SAS software was used to perform an analysis of variance (ANOVA) using the PROC MIXED model. Each site × year was considered one environment. All factors and their interactions were random effects.

## Results

### Seedling and APR genes

Gene postulation accompanied by molecular marker data were applied to determine seedling resistance genes in the WC. The ITs of the 30 differential lines to the ten races of *P. triticina* are shown in Table 1. A total of 14 seedling genes were postulated: *Lr1*, *Lr2a*, *Lr2c*, *Lr3*, *Lr3ka*, *Lr9*, *Lr10*, *Lr14a*, *Lr14b*, *Lr15*, *Lr20*, *Lr26*, *Lr28* and *Lr30* (Supplementary data Table S3). Of those, *Lr20* was postulated in 86 accessions, *Lr3* in 62, *Lr28* in 19, *Lr15* in 16, *Lr14a* in 12, *Lr2c* in four and *Lr30* and/or *Lr3ka* in two accessions, while *Lr2a*, *Lr9*, *Lr14b* and *Lr26* were each postulated in a single accession (Supplementary data Table S3). *Lr2a*, *Lr2b*, *Lr11*, *Lr16*, *Lr1*7, *Lr25*, *Lr3bg* and *LrB* were not postulated in any accessions because none of the accessions showed IT patterns similar to their corresponding differential lines. *Lr18*, *Lr19*, *Lr21*, *Lr29* and *Lr32* had LITs to the ten races and, as such, may not be present in the WC.

Based on this overall gene postulation, the collection was divided into three major groups. Group 1 contained 40 accessions that had HITs to all ten races and thus may not have any of the seedling resistance genes of the differential set. Group 2 contained 11 accessions with ITs that did not match any of the differential lines with known genes, thereby representing unidentified or potentially novel (N) *Lr* genes. The term unidentified refers to known genes that could not be postulated with our differential set of 30 NILs and race panel of ten but that could be postulated with additional differential lines and/or races. Group 3 contained the remaining 224 lines which may possess one to five seedling resistance genes.

Based on the number of genes, group 3 was further subdivided into four subgroups. Subgroup1 contained 72 accessions postulated to have a single *Lr* gene. Within this subgroup, 17 and 15 accessions possessed *Lr1* and *Lr10*, respectively. Twenty-four accessions were negative for *Lr1* and *Lr10* but showed LIT to BBBD and were consequently postulated to have *Lr3*. Thirteen accessions had a LIT to race TDBG and hence were postulated to have *Lr20* alone. Accessions Janetzkis Fruher S and Prospur were postulated to only have *Lr28* and Sunbird was postulated to only have *Lr9* (Supplementary data Table S3). Subgroup 2 contained 96 accessions that possessed combinations of two seedling resistance genes including *Lr1*, *Lr2c*, *Lr3*, *Lr10*, *Lr14a*, *Lr15*, *Lr20*, *Lr26* and *Lr28* as well as unidentified or novel (*N*) genes. Fifty accessions were postulated to have two of these known genes and 46 accessions had one known gene and one unidentified or novel gene. Subgroup 3 included 44 accessions with three genes, of which six had three known genes while 38 had combinations of known and unidentified or novel genes. Subgroup 4 encompassed 12 accessions with more than three genes including ten accessions with four genes and two with five (Supplementary data Table S3).

Of the postulated or identified genes, *Lr1* and *Lr10* were the most frequent in North America and Asia, while in South America *Lr3*, *Lr10* and *Lr20* were prevalent. In Africa, *Lr1*, *Lr10* and *Lr20* were the most frequent. In Europe, *Lr1*, *Lr3*, *Lr10* and *Lr20* were found at higher frequency while in Oceania *Lr10* and *Lr20* were more frequent (Table 2). *Lr1* was more frequent in North America (18/36) followed by Africa (10/25) and South America (7/45). *Lr3* was found at higher frequency in South America (16/45) followed by Asia (16/48), and was present in a single accession from Africa. *Lr10* occurred at higher frequency in Africa (10/25) followed by Oceania (10/29) and Asia (4/48). *Lr20* dominated in African accessions (15/25) followed by South American ones but was found at very low frequency in North American germplasm (3/36) (Table 2).

**Table 2:** Geographical distribution of seedling resistance genes in the world collection of wheat

Continent	No. of accessions	*Lr1*	*Lr2a*	*Lr2c*	*Lr3*	*Lr3ka*	*Lr9*	*Lr10*	*Lr14a*	*Lr14b*	*Lr15*	*Lr20*	*Lr26*	*Lr28*	*Lr30*
North America	36	18	1	2	7	2	0	12	3	0	5	3	0	4	2
South America	45	7	0	1	16	0	0	11	5	0	3	20	0	5	0
Asia	48	12	0	0	16	0	0	4	1	1	4	7	1	0	0
Africa	25	10	0	0	1	0	0	10	1	0	0	15	0	1	0
Europe	65	16	0	0	12	0	0	15	1	0	1	20	0	4	0
Oceania	29	7	0	0	5	0	1	10	1	0	0	11	0	1	0
Unknown	27	4	0	1	5	0	0	8	0	0	3	10	0	4	0
Total	275	74	1	4	62	2	1	70	12	1	16	86	1	19	2

### Molecular marker analysis

Gene-specific markers to the seedling resistance genes *Lr1*, *Lr10* and *Lr21* were utilized to validate the gene postulation in the WC (Supplementary data Table S3) and to assess their potential as molecular screening tools. The *Lr1* and *Lr10* markers were present in 74 and 69 accessions, respectively, including 24 accessions that had both markers, while the *Lr21* marker was not present in the collection (Supplementary data Table S3). The *Lr1* and *Lr10* markers validated the gene postulation in the majority of the lines but 51 accessions possessing one and/or the other marker showed unexpected HITs to race BBBD (Supplementary data Table S3). The APR gene *Lr34* was assessed in the WC using the three gene-specific markers caSNP4, caIND11 and caSNP12 (Dakouri et al. 2010) and 52 accessions were positive for the *Lr34* markers (Supplementary data Table S3).

### Field resistance

**Fig. 1:**
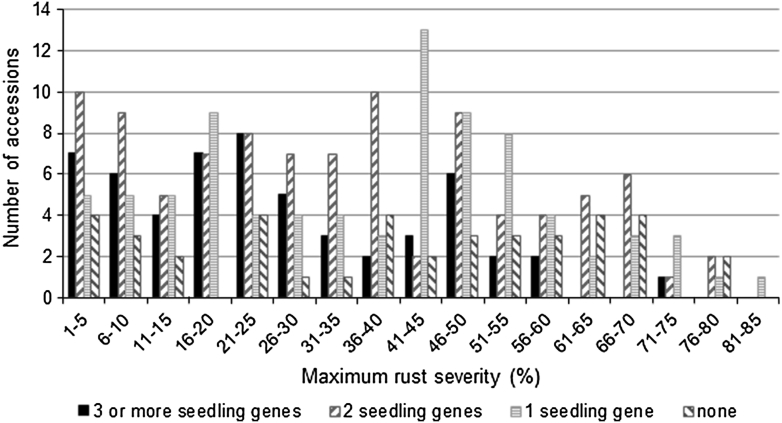
Frequency distribution of accessions plotted against maximum rust severity (MRS) based on the number of seedling resistance genes. Accessions with three or more genes had lower rust severity than accessions with zero, one or two seedling resistance genes

**Fig. 2:**
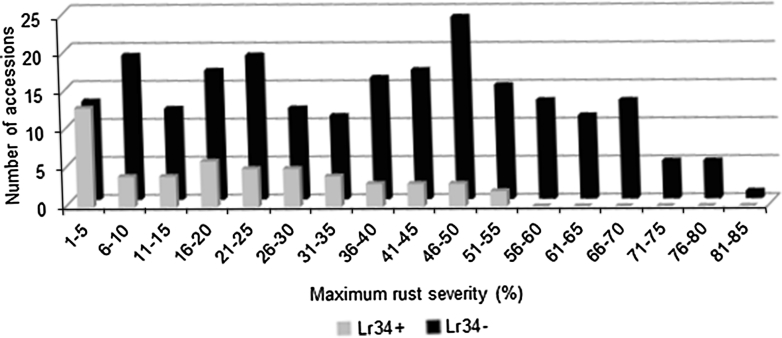
Frequency distribution of accessions plotted against maximum rust severity (MRS) based on the presence or absence of *Lr34* shows lower MRS in *Lr34*+ accessions

The WC was also evaluated for field resistance at three locations over 3 years. The ANOVA showed significant differences (*P* < 0.05) between accessions and between environments (Supplementary data Table S4). Significant differences also were observed between accessions with *Lr34* and those without *Lr34* as well as between accessions within the *Lr34*+ and *Lr34*− groups. The averaged maximum rust severity (MRS) and host response (HR) ranged from ~1R to 84S. Overall ~17 % of the accessions were considered highly resistant to leaf rust with MRS <10 %, ~15 % were resistant with MRS ranging from 10 to 20 %, ~28 % were moderately resistant with MRS between 20 and 40 %, 16 % moderately susceptible with MRS of 40–50 %, 20 % susceptible with MRS of 50–70 % and 5 % highly susceptible with MRS >70 %. Host response was not entirely correlated with MRS, and some accessions, for example Albimonte, had very low MRS but large pustules. In general, accessions possessing three or more genes did not have high severity ratings as compared to those having zero, one and two seedling resistance genes (Fig. 1). The average severity rating for the accessions with three or more seedling genes was 26 while it was 33 for those having two seedling genes, 38 for those with a single seedling gene and increased to 40 for the ones having none. Similarly, accessions containing *Lr34* were more resistant to leaf rust than those lacking it, regardless of their gene complement (Fig. 2). Of the 52 accessions with *Lr34*, 41 had an average MRS ranging from 1 to 35 %, which was similar to RL6058, a Thatcher NIL with *Lr34*. The other *Lr34*+ accessions showed MRS ranging from 36 to a maximum of 53 %.

Group 1 accessions, i.e., accessions without identified seedling resistance genes, displayed MRS ranging from 2 to 78 %. Seven group 1 accessions were classified as nearly immune or highly resistant with an average MRS ranging from 2 to <10 % and a resistant (R) to moderately susceptible (MS) reaction type (Supplementary data Table S3; Fig. 1). Six group 1 accessions possessed *Lr34* and exhibited MRS of 2–35 %. Group 2 with unidentified or N genes showed rust reading ranging from 1R to 76S. Accessions CN99032, Preludio and CN12624 were rated nearly immune or highly resistant, with rust reading 1R, 3RMR and 7RMR, respectively (Supplementary data Table S3). Within group 3, accessions possessing the gene combinations *Lr1* + *Lr26* and *Lr28* + *N* were particularly highly resistant to leaf rust with MRS <10 %. Accessions containing *Lr2c* + *N*, *Lr14a* + *N*, *Lr20* + *N*, *Lr28* + *N*, *Lr1* + *Lr20* + *N*, *Lr1* + *Lr14a* + *N*, *Lr10* + *Lr14a*, *Lr1* + *Lr20* + *Lr28* + *N* and *Lr1* + *Lr14a* + *Lr15* + *Lr20* + *N* in combination with *Lr34* were highly resistant when compared to other gene combinations.

**Fig. 3:**
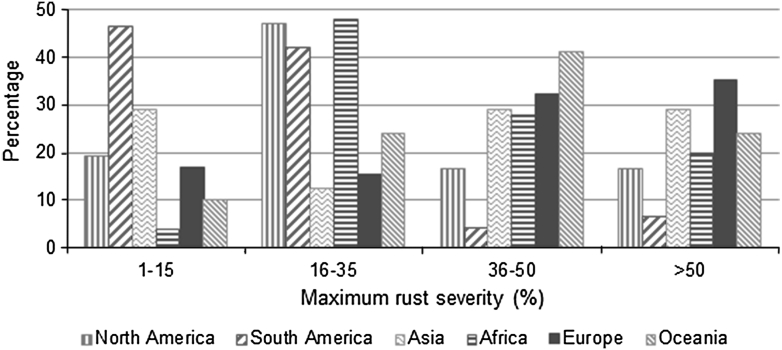
Frequency distribution of accessions plotted against maximum rust severity (MRS) based on their geographical distribution

At the geographical level, the average MRS was the lowest in South America (18 %) and the highest in European (42 %) and Oceanian germplasm (40 %). The majority of South American accessions (21/45) had an MRS from 1–15 %, while the proportion of highly resistant germplasm was much lower in the other continents and the lowest in Africa (1/25) within the same MRS range (Fig. 3). In Africa and North America, the majority of the accessions displayed an MRS ranging from 16 to 35 %. European accessions (23/65) had the highest percentage of susceptible lines with MRS over 50 %.

## Discussion

*Puccinia triticina*, the pathogen causing leaf rust, is present in most of the wheat-growing areas. Genetic resistance remains the most effective, economical and environmentally friendly strategy for controlling the negative impact of this disease. A survey of the seedling and APR genes in a world collection of wheat accessions using molecular markers and gene postulation accompanied by field resistance evaluations at three locations during 3 years is reported.

### Seedling and APR genes

Gene postulation has been widely applied as a quick method for hypothesizing the seedling resistance gene composition of germplasm. This method follows the gene-for-gene specificity theory that was first described by Flor (1942). Comparison of infection type profiles of unknown genotypes to various rust races with those of Thatcher near-isogenic lines, each with a single known gene, forms the basis of the gene postulation. However, this method does not enable differentiation between genes that have similar infection type profiles, e.g., *Lr1*, *Lr3* and *Lr10*. Considering the minimum number of genes required to explain phenotypes (McVey and Long 1993; Singh 1993b), gene postulation and molecular marker analyses of the WC identified 14 known seedling resistance genes present either as single genes or in combinations: *Lr1*, *Lr2a*, *Lr2c*, *Lr3*, *Lr9*, *Lr10*, *Lr14a*, *Lr14b*, *Lr15*, *Lr20*, *Lr26*, *Lr28* and *Lr3ka* and/or *Lr30*.

*Lr1*, *Lr3*, *Lr10* and *Lr20* were the most common genes in the WC, but their distribution across the globe varied. The first three were also the most common in American soft red winter wheat (Roelfs et al. 2000; Wamishe and Milus 2004). *Lr20* was the most frequent gene in Ethiopian wheat germplasm (Mebrate et al. 2008) but was found at low frequency in British wheat (Singh et al. 2001) and was rare in Australian germplasm, the American soft red winter wheat and Argentinian germplasm (Singh et al. 2007; Vanzetti et al. 2011; Wamishe and Milus 2004). A high *Lr1* frequency was previously reported in American hard red spring wheat, Australian wheat cultivars, Indian and Pakistani wheat, Mexican bread wheat and Chinese cultivars (Oelke and Kolmer 2004; Singh et al. 1999, 2007; Singh and Gupta 1991; Singh and Rajaram 1991) but was rare in Argentinian germplasm (Vanzetti et al. 2011). *Lr10* was commonly postulated in international winter wheat nurseries, Indian and Pakistani wheat cultivars, Mexican bread wheat, Brazilian and Argentinian wheat cultivars (McVey 1992; Singh and Gupta 1991; Singh and Rajaram 1991; Vanzetti et al. 2011; Zoldan and Barcellos 2002). Conversely, *Lr2a* and *Lr9* were rare in the WC with one accession each but were common in American soft red winter wheat (Roelfs et al. 2000; Wamishe and Milus 2004) and in cultivars from the UK (Singh et al. 2001). Located on the 1BL.1RS translocation, *Lr26* was reported at a high frequency in Chinese wheat germplasm (Singh et al. 1999), Argentinian cultivars (Vanzetti et al. 2011) and British cultivars (Singh et al. 2001) but was present in only a single accession (NING 8331) of the WC. The WC may simply have a low frequency of the rye translocation, a feature we did not evaluate.

Accessions El Gaucho, Bage, AC Minto, Americano 44D and Biggar, postulated to have *Lr2c* + *N*, *Lr1* + *10* + *20* + *N*, *Lr28* + *N*, *Lr1* + *10* + *N* and *Lr3* + *15* + *N*, respectively, were previously reported to possess *Lr11*, *Lr3bg*, *Lr11* + *13* + *21* + *22a*, *Lr13* + *34* and *Lr14a* + *13* in the same order (Kolmer 1994; Long and Kolmer 1989; Roelfs 1988). The *N* gene(s) in these accessions could be known gene(s) not in the differential sets, such as *Lr13* or *Lr22a*, or truly novel gene(s).

*Lr19*, *Lr21*, *Lr29* and *Lr32* displaying LITs to all ten races have not been deployed in wheat varieties (McIntosh et al. 1995; Mebrate et al. 2008; Wamishe and Milus 2004), and so their absence in the WC is not surprising. The same can be said for *Lr36* and *Lr38*. *Lr3ka* and/or *Lr30* were postulated in accessions Katepwa and Burnside but discrimination between the two genes was precluded by the lack of differentiating race(s).

The ten races and the 30 differential lines used in this study permitted gene postulation of 14 seedling resistance genes but these races and differential lines were not sufficient to postulate and determine the identity of all known resistance genes, indicative of some of the limitations of the gene postulation method (Hysing et al. 2006; Mebrate et al. 2008). A total of 96 accessions possessed combinations of known and unidentified (*N*) seedling resistance genes and 11 accessions were postulated to only have one or more *N* genes. The unidentified but known genes could be *Lr13*, *Lr27* + *Lr31* (Browder 1980; Dyck 1992; Pretorius et al. 1984) or not yet described alleles of known genes (Wamishe and Milus 2004). Allelism tests are appropriate alternatives but they are lengthy to perform (McCallum et al. 2010). Developing molecular markers tightly linked and, ideally, perfectly linked to genes and alleles of interest is the method of choice for determining the identity of known genes (Tanksley et al. 1989).

Variability in the LIT scores (i.e., 0, ;, 1; 2, 22+) is a common indication of heterozygosity for avirulence among *P. triticina* isolates (Samborski and Dyck 1968; Wamishe and Milus 2004). Temperature sensitivity is another possible cause of LIT score variability such as previously reported for *Lr11* and *Lr18* (Long and Kolmer 1989; McIntosh et al. 1995). Temperatures as low as 17 °C were used to postulate *Lr11* when tested with homozygous avirulent races (Wamishe and Milus 2004). In our study, all races were tested within a temperature range of 18–22 °C, which might explain the lack of detection of *Lr11*.

### Field resistance

To better understand the effect of seedling and APR genes on leaf rust under field conditions, the WC was evaluated in the field at three locations over 3 years. Field data showed that 60 % of the accessions were highly to moderately resistant to leaf rust with MRS of 1–40 %, while 40 % were moderately to highly susceptible with MRS greater than 40 %.

Overall, the rust severity decreased as the number of seedling resistance genes increased, indicating the importance of gene pyramiding in reducing the damage caused by leaf rust including yield loss (Šliková et al. 2004). Gene pyramiding, a process of combining more than one resistance gene in a single genotype, has been a proven strategy for developing long-lasting resistance to plant disease (Singh et al. 2000). This strategy has been exploited for resistance to bacterial blast and bacterial blight in rice (Hittalmani et al. 2000; Huang et al. 1997), powdery mildew and leaf rust in wheat (Liu et al. 2000; Šliková et al. 2004) and stripe rust in barley (Castro et al. 2003). In our study, accessions of the WC contained one to five genes and, in total, 43 gene combinations were observed including 19 two-gene, 19 three-gene, 6 four-gene and 2 five-gene combinations. Accessions possessing three genes or more clearly displayed an overall lower rust severity rating compared to those with zero, one or two genes (Fig. 1).

Subgroup1 is composed of accessions with one seedling resistance gene including *Lr1*, *Lr3*, *Lr9*, *Lr10*, *Lr20* and *Lr28* that have already been overcome (Kolmer 1996). Within this subgroup, correlations between the gene content and low MRS were not consistent, which indicates that low levels of MRS in some accessions cannot solely be accounted for by the presence of these genes alone but possibly by the presence of one or more APR genes (Kolmer 1996). *Lr20* did not contribute significantly to leaf rust resistance (McIntosh et al. 1995; Wamishe and Milus 2004). The same conclusion may apply to *Lr1*, *Lr3*, *Lr10* and *Lr28*.

Subgroup 2 comprised accessions with two genes each. Two-gene combinations also provided variable levels of MRS ranging from 2 to 78 %. Accessions possessing *Lr28* + *N* consistently showed low levels of MRS ranging from 3 to 17 %, indicating the potential of this combination in leaf rust resistance. Accessions El Gaucho and NING 8331with *Lr2c* + *N* and *Lr1* + *Lr26*, respectively, were highly resistant with MRS of 2 and 3 % and may also represent good combinations for improved leaf rust resistance.

Despite the fact that most combinations within subgroup 3 provided high to moderate levels of resistance, the gene combination *Lr1* + *Lr10* + *N* seems to provide better levels of resistance. The observed resistance level may be solely accounted for by the *N* gene(s) because *Lr1* and *Lr10* have been overcome (Kolmer 1996). The only exception was accession Kenya which had a moderately susceptible phenotype accompanied by a HIT to BBBD.

Subgroup 4 with four and five seedling resistance genes was overall more resistant than the other subgroups. The best levels of resistance were observed for combinations with *N* gene(s), stressing once more the need to further investigate these genes.

*Lr34* has been the most important leaf rust resistance gene identified to date because of its race non-specificity, its durability, its synergistic and pleiotropic effects and its positive effect on yield (Dyck 1987, 1991; Dyck et al. 1994; McCallum et al. 2007; McIntosh 1992; Samborski 1985; Singh 1992a, b, 1993a; Singh and Huerta-Espino 1997; Spielmeyer et al. 2005). The majority of *Lr34*+ accessions (79 %) had high to moderate levels of resistance with MRS ranging from trace to 35 %, similar to RL6058 (i.e., Tc-*Lr34* NIL). The remaining eleven *Lr34*+ accessions (~21 %) showed average MRS ranging from 36 to 52 %. The specific reason(s) for the relatively higher rust severity in these *Lr34*+ accessions can only be speculated. DNA methylation was reported to play a role in the expression of APR genes in rice, where a correlation was observed between hypermethylation and repression of gene expression (Sha et al. 2005). Recently, Wu et al. (2012) reported the presence of a stripe rust (*Yr18* function) inhibitor to *Lr34*/*Yr18*/*Pm38* in some Chinese landraces. Such inhibitors were not identified in breeding lines or for the *Lr34* leaf rust function, but this too suggests the possibility of functional inhibitors to another *Lr* gene. Population development, gene expression, sequencing and methylation pattern analyses are required to investigate these hypotheses.

The interactions between *Lr34* and other leaf rust resistance genes including seedling and APR genes were described in previous reports and also herein. Here, we highlighted gene combinations that have provided excellent levels of resistance in our multi-year, multi-location field trials. The relatively high levels of resistance in *Lr34*+ accessions with no seedling resistance genes might be explained by the presence of additional APR genes (Li et al. 2010) such as *Lr12*, *Lr13*, *Lr22a*, *Lr46*, *Lr67* and *Lr68*, or novel APR genes. Synergy between *Lr34* and other APR genes was previously reported (Kloppers and Pretorius 1997; Sawhney 1992). Cultivars possessing combinations of *Lr34* + *Lr12* (e.g., Chinese Spring) and *Lr34* + *Lr13* (e.g., Roblin) were highly resistant with 5RMR and 10MRMS rust reading, respectively (Dyck 1991, 1992). Accession El Gaucho which had the gene combination *Lr34* + *Lr2c* + *N* was highly resistant with a severity of 2 % and an RMR reaction type, indicative of the potential synergy between these genes. Seven accessions had the combination of *Lr3* + *Lr34* with rust rating ranging from 1RMS to 48MSS. Because *Lr3* is defeated, additional APR genes are hypothesized in accessions with the lowest MRS. *Lr9*, previously reported as an important leaf rust resistance gene in soft red winter wheat (Kolmer 2003), was only postulated in Sunbird and, in combination with *Lr34*, provided only a moderate level of field resistance to leaf rust.

The high levels of resistance in some accessions without *Lr34* may be caused by various gene combinations not fully characterized here, including other APR genes. *Lr13*, which originated from South American germplasm, was commonly found in wheat germplasm worldwide (McIntosh et al. 1995; Singh et al. 1998, 2001). Despite being defeated, *Lr13* in combination with other APR genes may provide an acceptable level of field resistance (Kolmer 1992). *Lr12* is also frequent in wheat cultivars from Australia, China, North America and South America (Kolmer 2003; McIntosh et al. 1995; Park and McIntosh 1994; Wamishe and Milus 2004). The WC may contain some or all known APR genes as well as novel genes, alone or in combinations. The accession Kanred, for example, had no seedling resistance genes but displayed a low severity of 5 % and a host response (HR) of R in two locations and MS in one, indicating the presence of unidentified genes. The variability in host response (i.e., R in two locations and MS in one) could be due to environmental effects.

The majority of South American accessions had multiple resistance genes, both seedling and APR, combined with unidentified (*N*) genes, which might explain the low level of rust severity in these accessions. In Europe and Oceania, on the other hand, most accessions had either zero or one seedling gene, thus explaining the high level of rust severity in these accessions. This may indicate intensive breeding programs for leaf rust resistance in South America as compared to the other areas of the world.

### Molecular marker analysis

Molecular markers have been invaluable tools in plant breeding, including gene identification and marker-assisted selection. One of the major applications of markers in breeding for leaf rust resistance is to determine the number and identity of the resistance genes in cultivars or germplasm. Markers linked to *Lr1*, *Lr3a*, *Lr9*, *Lr10*, *Lr13*, *Lr14a*, *Lr16*, *Lr17a*, *Lr19*, *Lr20*, *Lr21*, *Lr22a*, *Lr23*, *Lr24*, *Lr25*, *Lr26*, *Lr27*, *Lr28*, *Lr29*, *Lr31*, *Lr32*, *Lr34*, *Lr35*, *Lr37*, *Lr38*, *Lr39*, *Lr42*, *Lr46*, *Lr47*, *Lr48*, *Lr49*, *Lr50*, *Lr51*, *Lr52*, *Lr56*, *Lr57*, *Lr58*, *Lr60*, *Lr61*, *Lr63*, *Lr66* and *Lr67* have been published [reviewed in McCallum et al. (2012)] and, since then, markers for *Lr68* (Herrera-Foessel et al. 2012) have also been reported. While these markers are linked to *Lr* genes, they are not all perfectly diagnostic. Their usefulness depends mainly on the genetic background, the tightness of the linkage, whether the gene is from an alien source, the polymorphism level and the robustness of the markers. Gene-specific markers are more accurate but, as we discovered for *Lr1* and *Lr10*, they may not correlate perfectly with the phenotype. This was also observed for *Lr1* (Da Silva et al. 2008), although in this case they were not using a gene-specific marker (Feuillet et al. 1995). Our intention was to use the molecular markers to provide validation for the gene postulation and to demonstrate their usefulness as screening tools for breeders and pathologists.

While the majority of the accessions postulated through seedling tests to have *Lr1* and/or *Lr10* were validated by their respective markers, 51 accessions, although possessing the *Lr1* and/or *Lr10* gene-specific markers, had HITs to leaf rust race BBBD. Many *Lr10* haplotypes have been described but only one was reported to confer resistance (Sela et al. 2012). Such extensive haplotype description has not been done for *Lr1*. Even though the *Lr1* marker is gene-specific, it may not be perfectly diagnostic because the possibility of additional functional and non-functional haplotypes cannot be ruled out. In the case of *Lr10*, *RGA2*, a gene closely linked to *Lr10*, has been shown to be necessary for its function (Loutre et al. 2009). Indeed, some *R* genes can only act in pairs, but, in addition, NBS-LRR-driven resistance can be mediated through several different resistance mechanisms (Eitas and Dangl 2010). Finally, studies on *Lr26* and *Pm8* reported their non-functionality in the presence of inhibitor genes (Hanušová et al. 1996; Li et al. 2010). Even though inhibitor genes have not been reported to date for *Lr1* and *Lr10*, this mechanism, the dual gene action and the potential for undescribed functional and non-functional haplotypes may all explain the discrepancies between the marker data and the phenotypic results with BBBD.

*Lr21* originated from the diploid wheat species *Aegilops tauschii* and was first introgressed into wheat cultivar Thatcher (Rowland and Kerber 1974). This gene was not present in the WC, indicating its limited deployment to date in breeding programs.

Our results clearly demonstrate the usefulness of gene-specific markers such as *Lr1* (Cloutier et al. 2007), *Lr10* (Schachermayr et al. 1997), *Lr21* (Huang and Gill 2001) and *Lr34* (Krattinger et al. 2009; Lagudah et al 2009; Dakouri et al. 2010) in gene postulation. Gene-specific markers have the potential to be highly diagnostic but they are not always perfect. Extensive haplotyping within the primary and ideally the secondary and tertiary gene pools as well as in evolutionarily related species such as rye, barley and *Brachypodium* is necessary to provide a comprehensive understanding of the gene(s) and for the development of one or a suite of perfectly diagnostic markers.

APR is conferred by genes that are effective at the adult plant stage. They are mostly race non-specific and provide similar levels of resistance to all leaf rust races (Das et al. 1993; Ohm and Shaner 1976). Using three gene-specific markers (Dakouri et al. 2010), the APR gene *Lr34* was identified in 52 of the 275 accessions of the WC. This gene has been widely utilized in breeding for leaf rust resistance and is commonly found in world wheat germplasm (Kolmer et al. 2008), particularly in Asian and South American germplasm (Singh et al. 1999). Molecular markers are especially useful for APR genes because their phenotyping is challenging. APR phenotypes are better characterized under field conditions and the few indoor tests (e.g., cold temperature test) are lengthy (Krattinger et al. 2009). The *Lr34* marker caSNP12 was perfectly diagnostic for 700 lines (Dakouri et al. 2010) but it is not ideal as a dominant marker because failed reactions and negative markers are confounded. Marker caIND11 constitutes an excellent alternative, being co-dominant and with a diagnostic rate of 99.7 % (Dakouri et al. 2010).

## Conclusions

Efficient exploitation of genetic resistance to leaf rust demands a detailed examination of the occurrence and distribution of both seedling and APR genes. Using molecular markers combined with gene postulation, 14 seedling resistance genes and one APR gene were determined to be present in a world collection of wheat. Additional seedling and APR genes may be present but could not be postulated based on the differential lines, leaf rust races and diagnostic molecular markers. The world collection is a potential reservoir for several novel resistance genes, both seedling and APR. The majority of these accessions would have multiple genes, with some of them providing excellent levels of resistance. However, the actual number of genes contained in each accession can only be determined by genetic analysis. The work presented herein provides further evidence to support the claim that *Lr34* is the single most significant and durable gene in breeding programs for leaf rust resistance. The results of this study highlight the potential and limitations of molecular markers for leaf rust gene postulation and the importance of gene pyramiding to provide acceptable long-term resistance to wheat leaf rust with emphasis on the important role of APR genes.

## Electronic supplementary material

Below is the link to the electronic supplementary material.

Supplementary material 1 (PDF 1,046 kb)
