# Supplementary material for: Molecular and phenotypic characterization of seedling and adult plant leaf rust resistance in a world wheat collection
Source: Mol Breed. 2013 Aug 11;32(3):663–77. doi: 10.1007/s11032-013-9899-8 (PMC3782647; doi:10.1007/s11032-013-9899-8)
Supplement: Supplementary file 1 — Supplementary material 1 (PDF 1,046 kb) [file 11032_2013_9899_MOESM1_ESM.pdf]

**Table S1**. Accession names, country of origin and status of the WC germplasm

| Accession | Country of Origin | Status |
|---|---|---|
| North America | | - |
| AC Minto | Canada | Released cultivar |
| AC Vista | Canada | Released cultivar |
| Biggar | Canada | Released cultivar |
| Bluesky | Canada | Released cultivar |
| Burnside | Canada | - |
| BW 90 | Canada | - |
| CDC Teal | Canada | Released cultivar |
| Columbus | Canada | Released cultivar |
| Corrine | Canada | - |
| Glenavon | Canada | - |
| HY 320 | Canada | Released cultivar |
| HY358 | Canada | Released cultivar |
| Katepwa | Canada | Released cultivar |
| Laura | Canada | Released cultivar |
| Neepawa | Canada | Released cultivar |
| Oslo | Canada | Released cultivar |
| RL4452 | Canada | Breeding line |
| Wildcat | Canada | Released cultivar |
| Roblin | Canada | Released cultivar |

| **Accession** | **Country of Origin** | **Status** |
|---|---|---|
| Arcola | Canada | Released cultivar |
| Santa Elena | Mexico | Released cultivar |
| Bajio | Mexico | Released cultivar |
| Lom | Mexico | Released cultivar |
| Tobari F66 | Mexico | Released cultivar |
| Andes | Mexico | Released cultivar |
| Beloglina | USA | Released cultivar |
| Marquillo | USA | Released cultivar |
| Carina | USA | Released cultivar |
| Touse | USA | Released cultivar |
| Hope | USA | Released cultivar |
| Kanred | USA | Released cultivar |
| Nordic | USA | Released cultivar |
| Prospur | USA | Released cultivar |
| Red River 68 | USA | Released cultivar |
| Stoa | USA | Released cultivar |
| Wheaton | USA | Released cultivar |
| Buffum | USA | - |
| South America | | - |
| Argentina | Argentina | - |
| Benvenuto 3085 | Argentina | Released cultivar |

| Accession | Country of Origin | Status |
|---|---|---|
| Buck Nandu | Argentina | Released cultivar |
| Buck Pucara | Argentina | Released cultivar |
| Calidad | Argentina | Released cultivar |
| El Gaucho | Argentina | Released cultivar |
| Klein otto Wulff | Argentina | Released cultivar |
| Klein Sendero | Argentina | Released cultivar |
| Klein Credito | Argentina | Released cultivar |
| Klein Toledo | Argentina | Breeding line |
| Klein Universal | Argentina | Released cultivar |
| Retacon INTA | Argentina | Released cultivar |
| Rio Negro | Argentina | Released cultivar |
| Tezanos Printos Precoz | Argentina | Released cultivar |
| Universal II | Argentina | Released cultivar |
| Victoria INTA | Argentina | Released cultivar |
| Parana | Argentina | - |
| PAMPA-INTA | Argentina | Released cultivar |
| Olaeta Calandria | Argentina | Released cultivar |
| Sinvalocho | Argentina | Released cultivar |
| Cinquentenario | Brazil | Released cultivar |
| Colonias | Brazil | Released cultivar |
| Encruzilhada | Brazil | Released cultivar |

| Accession | Country of Origin | Status |
|---|---|---|
| Fortaleza | Brazil | Released cultivar |
| Frontana | Brazil | Released cultivar |
| Fronteira | Brazil | Released cultivar |
| Preludio | Brazil | Released cultivar |
| Surpresa | Brazil | Released cultivar |
| Toropi | Brazil | Released cultivar |
| Trintecinco | Brazil | Released cultivar |
| Tropeano | Brazil | Released cultivar |
| Bage | Brazil | Released cultivar |
| Combate | Brazil | Released cultivar |
| Trintani | Brazil | Released cultivar |
| Bola Picota | Colombia | Released cultivar |
| Bonza | Colombia | Released cultivar |
| CN12624 | Colombia | - |
| Menkemen | Colombia | Released cultivar |
| Atacatzo 1 | Ecuador | - |
| CN11698 | Peru | - |
| CN12268 | Peru | - |
| CN12358 | Peru | - |
| Helvia | Peru | Released cultivar |
| Maribal 50 | Peru | - |

| Accession | Country of Origin | Status |
|---|---|---|
| Americano 44D | Uruguay | Released cultivar |
| Asia | | - |
| Chengdu Guangtou | China | Landrace |
| Fengcheung No. 1 | China | - |
| Fu Mai No 3 | China | Released cultivar |
| Ho chun No. 12 | China | - |
| Hsin ShuKuan#1 | China | - |
| Li Yang Wong Shu Bai | China | Released cultivar |
| Nan Tong DA Huang PI | China | Released cultivar |
| NING 7840 | China | - |
| NING 8026 | China | - |
| NING 8331 | China | Breeding line |
| Shu Chou Wheat #3 | China | Released cultivar |
| Su Mai No 3 | China | Released cultivar |
| Xiang Mai No 1 | China | Released cultivar |
| Yang Mai No.1 | China | Released cultivar |
| Chinese Spring | China | Landrace |
| Girija | India | Released cultivar |
| Kenphad 25 | India | Released cultivar |
| New Pusa | India | - |
| Sonalika | India | Released cultivar |

| Accession | Country of Origin | Status |
|---|---|---|
| Vijay | India | Released cultivar |
| Surkh | India | Landrace |
| Azar | Iran | Released cultivar |
| Rayhany | Iran | - |
| Shahpassand | Iran | - |
| Ajelea | Iraq | - |
| Gogatsukomugai | Japan | Released cultivar |
| Ikuzai #1 | Japan | Released cultivar |
| Nobeoka Bozu | Japan | Landrace |
| Norin 75 | Japan | Released cultivar |
| Nyu Bay | Japan | Landrace |
| Shinchunaga | Japan | Landrace |
| CN11204 | Jordan | - |
| Golubka | Kazakhstan | Released cultivar |
| karagandinskaja | Kazakhstan | Released cultivar |
| Koktunkulskaja 332 | Kazakhstan | - |
| Krasnaja Svenzda | Kazakhstan | - |
| Ferrugineum-87 | Kyrgystan | - |
| CN29854 | Nepal | - |
| CN29856 | Nepal | - |
| CN29858 | Nepal | - |

| Accession | Country of Origin | Status |
|---|---|---|
| CN29871 | Nepal | - |
| CN42522 | Nepal | - |
| CN10241 | Pakistan | - |
| CN12251 | Pakistan | - |
| CN30213 | Pakistan | - |
| CN9934 | Pakistan | - |
| CN9936 | Pakistan | - |
| 71GN No 115 | Syria | - |
| Africa | | - |
| CN12666 | Congo | - |
| CN11720 | Egypt | - |
| CN12425 | Egypt | - |
| CN9794 | Egypt | - |
| Giza 141 | Egypt | Breeding line |
| Camadi | Ethiopia | Landrace |
| CN11307 | Ethiopia | - |
| CN6024 | Ethiopia | - |
| CN6025 | Ethiopia | - |
| CN6030 | Ethiopia | - |
| CN10661 | Kenya | - |
| Equator | Kenya | Released cultivar |

| Accession | Country of Origin | Status |
|---|---|---|
| CN10750 | Kenya | - |
| CN12388 | Kenya | - |
| Cobbs 1066 | Kenya | Released cultivar |
| Kenya 58 | Kenya | Released cultivar |
| Kenya Farmer | Kenya | Released cultivar |
| Kenya Governor | Kenya | Released cultivar |
| Kenya | Kenya | Breeding line |
| Daeraad | South Africa | Released cultivar |
| Dromedaris | South Africa | Released cultivar |
| Duiker | South Africa | Released cultivar |
| Penkop | South Africa | Released cultivar |
| Klein Trou | South Africa | Released cultivar |
| Koalisie | South Africa | Released cultivar |
| Europe | | - |
| CN11461 | Austria | - |
| Fruher Tiroler Bin | Austria | - |
| Alfy 2 | Belgium | Released cultivar |
| Fylby | Belgium | Released cultivar |
| Jufy 2 | Belgium | Released cultivar |
| Phoebus | Belgium | Released cultivar |
| CN40750 | Bulgaria | - |

| Accession | Country of Origin | Status |
|---|---|---|
| Minskaja | Bulgaria | - |
| Lada | Bulgaria | Released cultivar |
| Maja | CSK | Released cultivar |
| Sandra | CSK | Released cultivar |
| Vega | CSK | Released cultivar |
| KVL 2263 | Denmark | - |
| Etoile De Choisy | France | Released cultivar |
| Ventura | France | Released cultivar |
| Cargimarec | France | Released cultivar |
| Bersee | France | - |
| Hybride Du Jubile | France | - |
| Werna | France | Released cultivar |
| Prinqual | France | Released cultivar |
| Froubou | Germany | - |
| Heines Peko | Germany | Released cultivar |
| Janetzkis Fruher S | Germany | Released cultivar |
| Hohenhein | Germany | - |
| Janetzkis Probat | Germany | - |
| Strubes Fortschritt | Germany | Released cultivar |
| Funo | Italy | Released cultivar |
| Falerio | Italy | - |

| Accession | Country of Origin | Status |
|---|---|---|
| Mentana | Italy | Released cultivar |
| CN11642 | Poland | - |
| Magueija | Portugal | Landrace |
| Pirana | Potrugal | Released cultivar |
| Da Maia | Portugal | Landrace |
| Acadimia 48 | Romania | - |
| Dvina | Russia | Released cultivar |
| Gorkorskaja-15 | Russia | Released cultivar |
| Narinskajal | Russia | - |
| Pamjat Urala | Russia | - |
| Severodvinskaja 1 | Russia | Released cultivar |
| Udarnica | Russia | Released cultivar |
| Moskovka | Russia | Released cultivar |
| Irkutskaja 49 | Russia | - |
| Krasnokutka 3 | Russia | Unreleased cultivar |
| Onohoiskaja 4 | Russia | - |
| Ladoga | Russia | Landrace |
| Jakutjanka | Russia | Released cultivar |
| Krasnojarskaja 1103 | Russia | - |
| Pobeda | Russia | Released cultivar |
| Iskra | Russia | Released cultivar |

| Accession | Country of Origin | Status |
|---|---|---|
| Kamalinka | Russia | Released cultivar |
| Ladoga | Russia | Landrace |
| Albimonte | Switzerland | Released cultivar |
| Dominator | UK | Released cultivar |
| Holdfast | UK | Released cultivar |
| Little Joss | UK | Released cultivar |
| Red Marvel | UK | Released cultivar |
| Squarhead's Master | UK | Released cultivar |
| Warden | UK | Released cultivar |
| Musket | UK | Released cultivar |
| Tonic | UK | Released cultivar |
| Kinsman | UK | - |
| DART | UK | Released cultivar |
| Katja A1 | UK | Released cultivar |
| Setter | UK | Released cultivar |
| Odess kaja 13 | Ukraine | Released cultivar |
| Oceania | | - |
| Comeback | Australia | Released cultivar |
| Bunyip | Australia | Released cultivar |
| cailloux | Australia | Released cultivar |
| BAART | Australia | Released cultivar |

| Accession | Country of Origin | Status |
|---|---|---|
| Bungulla | Australia | Released cultivar |
| Chara | Australia | Released cultivar |
| Federation | Australia | Released cultivar |
| Gabo | Australia | Released cultivar |
| Hard Federation | Australia | Released cultivar |
| Hofed | Australia | Released cultivar |
| John Brown | Australia | Released cultivar |
| Jumbuck | Australia | Released cultivar |
| Kukri | Australia | - |
| Major | Australia | Released cultivar |
| Pacific Bluestem | Australia | Released cultivar |
| Sabre | Australia | Released cultivar |
| Sunstar | Australia | Released cultivar |
| White Federation | Australia | Released cultivar |
| Warigo | Australia | Released cultivar |
| CD87 | Australia | - |
| Bencubbin | Australia | Released cultivar |
| Sunbird | Australia | Released cultivar |
| Arawa | New Zealand | Released cultivar |
| CN12035 | New Zealand | - |
| Endeavor | New Zealand | - |

| **Accession** | **Country of Origin** | **Status** |
|---|---|---|
| Hilgendorf | New Zealand | Released cultivar |
| Oroua | New Zealand | Released cultivar |
| Otane | New Zealand | Released cultivar |
| Cross 7 | New Zealand | Released cultivar |
| Unknown | | |
| CN33802 | Unknown | - |
| CN11739 | Unknown | - |
| G3893 | Unknown | - |
| CWI42988 | Unknown | - |
| CN12361 | Unknown | - |
| Dorziyeh Karak | Unknown | - |
| CN99032 | Unknown | - |
| CN9940 | Unknown | - |
| Kostoff's Triple Hybrid | Unknown | - |
| CN12213 | Unknown | - |
| CN33892 | Unknown | - |
| CN33893 | Unknown | - |
| CN2758 | Unknown | - |
| CN11647 | Unknown | - |
| CN33803 | Unknown | - |
| CN32504 | Unknown | - |

| Accession | Country of Origin | Status |
|---|---|---|
| CN12442 | Unknown | - |
| CN1849 | Unknown | - |
| CN12261 | Unknown | - |
| Kozlodui | Unknown | - |
| Dorziyey Safra | Unknown | - |
| CN6171 | Unknown | - |
| Tambillo 1 | Unknown | - |
| Indur Compactum | Unknown | - |
| CN37599 | Unknown | - |
| CN12229 | Unknown | - |
| CN40895 | Unknown | - |

**Table S2.** Primer names, sequences, PCR conditions, amplicon sizes and references for four *Lr* gene specific markers

| Gene | Primer Name | Sequence | PCR condition | Resolution method | Amplicon sizes | Reference |
|---|---|---|---|---|---|---|
| ***Lr1*** | Glenlea5_F233<br>Glenlea5_R712 | AGTCTGCACAATCTTTTCCGG<br>ATCTGTAGTTGGTCCACCAAGG | Initial denaturation: 94˚C, 4 min<br>Denaturation: 94˚C, 15 sec<br>Annealing: 65˚C, 30 sec<br>Extention: 72˚C, 30 sec<br>Cycles: 35<br>Final extension: 72˚C, 10 min<br>End: 15˚C constant | 1.5% agarose gel | 500/N | Cloutier et al. 2007 |
| ***Lr10*** | Lrk10D1<br>Lrk10D2 | GAAGCCCTTCGTCTCATCTG<br>TTGATTCATTGCAGATGAGATCACG | Initial denaturation: 94˚C, 3 min<br>Denaturation: 94˚C, 45 sec<br>Annealing: 57˚C, 45 sec<br>Extention: 72˚C, 30 min<br>Cycles: 35<br>Final extension: 72˚C, 10 min<br>End: 15˚C constant | 1.5% agarose gel | 300/400/500 | Schachermayr et al. 1997 |

| Gene | Primer Name | Sequence | PCR condition | Resolution method | Amplicon sizes | Reference |
|---|---|---|---|---|---|---|
| ***Lr21*** | Lr21-227F<br>Lr21-550R | TATTTGACATCAGTCATTGAGG<br>CATCTTGAGATAGCAGTAAGG | Initial denaturation: 95˚C, 5 min<br>Denaturation: 95˚C, 1 min<br>Annealing: 54˚C,  I min<br>Extention: 72˚C, 1 min<br>Cycles: 34<br>Final extension: 72˚C, 10 min<br>End: 15˚C constant | 1.5% agarose gel | 400/N | Huang and Gill 2001 |
| ***Lr34*** | caIND11 | F: GTCTCCCAATCTGCATGCTC<br>R: TACCTCCCAAAAGCCAGTTG | Initial denaturation: 93˚C, 2 min<br>Denaturation: 94˚C, 1 min<br>Annealing: 58˚C, 50 sec<br>Extention: 72˚C, 1 min<br>Cycles: 30<br>Final extension: 72˚C, 10 min<br>End: 15˚C constant | ABI3130*x*1 | 394/397 | Dakouri et al. 2010 |

| Gene | Primer Name | Sequence | PCR condition | Resolution method | Amplicon sizes | Reference |
|---|---|---|---|---|---|---|
| | caSNP4 | F: GCGTTTCTGTCACCAGAAGT<br>R: AATAAACTCGCGCCTCTTGA | Initial denaturation: 94˚C, 5 min<br>Denaturation: 94˚C, 1 min<br>Annealing: 65˚C, 30 sec<br>Extention: 72˚C, 1 min<br>Cycles: 35<br>Final extension: 72˚C, 10 min<br>End: 15˚C constant | 1.5% agarose gel | 390/N | |
| | caSNP12 | F: TCCCCAGTTTAACCATCCTG<br>R: CATTCAGTCACCTCGCAGC | | | 234/N | |

**Table S3**. Seedling resistance gene postulation based on the infection types of 10 races of *P. triticina* and molecular data, and evaluation of maximum rust severity and host response of a world collection of 275 wheat accessions

| Accession name | Seedling genes | BBBD | MBDS | MGBJ | TJBJ | TDBG | MBRJ | PBDQ | THMJ | TNRJ | TCRJ | Marker data | | | MRS | SE | HR | | |
|---|---|---|---|---|---|---|---|---|---|---|---|---|---|---|---|---|---|---|---|
| | | | | | | | | | | | | *Lr1* | *Lr10* | *Lr34* | | | WPG | GLN | POR |
| Group1 | | | | | | | | | | | | | | | | | | | |
| Neepawa | none | 3 | 3 | 3 | 3 | 33+ | 33+ | 33+ | 3 | 33+ | 3 | | | | 61 | 4 | MRMS | S | S |
| Kanred | none | 3 | 3 | 3 | 3 | 3 | 3 | 33+ | 3 | 33+ | 3 | | | | 4 | 1 | R | MS | R |
| Chinese Spring | none | 3 | 3 | 3 | 3 | 3+ | 3 | 33+ | 3 | 33+ | 3 | | | *Lr34* | 2 | 1 | MS | ? | R |
| CN33802 | none | 3 | 3 | 3 | 3 | 4 | 33+ | 33+ | 3 | 3 | 3 | | | | 78 | 3 | ? | ? | MS |
| CN11739 | none | 3 | 3 | 3 | 3 | 33+ | 33+ | 33+ | 3 | 3 | 3 | | | | 70 | 4 | S | MSS | MSS |
| G3893 | none | 3 | 3 | 3 | 3 | 33+ | 33+ | 3 | 3 | 3 | 3 | | | | 30 | 5 | MR | MSS | S |
| CWI42988 | none | 3 | 3 | 3 | 3 | 33+ | 3 | 33+ | 3 | 3 | 3 | | | | 41 | 5 | MRMS | MS | R |
| Surkh | none | 3 | 3 | 3 | 3 | 33+ | 33+ | 3 | 3 | 3 | 3 | | | | 78 | 2 | S | S | S |
| Li Yang Wong Shu Bai | none | 3 | 3 | 3 | 3 | 33+ | 3 | 33+ | 3 | 3 | 3 | | | *Lr34* | 4 | 1 | MS | R | MS |
| Ajelea | none | 3 | 3 | 3 | 3 | 33+ | 33+ | 33+ | 3 | 3 | 3 | | | | 2 | 1 | ? | R | R |
| Nyu Bay | none | 3 | 3 | 3 | 3 | 33+ | 33+ | 33+ | 3 | 3 | 3 | | | *Lr34* | 14 | 3 | MS | MS | MS |
| CN29856 | none | 3 | 3 | 3 | 3 | 43+ | 33+ | 33+ | 3 | 3 | 3 | | | | 63 | 3 | MS | S | MS |

| Accession name | Seedling genes | BBBD | MBDS | MGBJ | TJBJ | TDBG | MBRJ | PBDQ | THMJ | TNRJ | TCRJ | Marker data | | | MRS | SE | HR | | |
|---|---|---|---|---|---|---|---|---|---|---|---|---|---|---|---|---|---|---|---|
| | | | | | | | | | | | | *Lr1* | *Lr10* | *Lr34* | | | WPG | GLN | POR |
| CN29854 | none | 3 | 3 | 3 | 3 | 33+ | 33+ | 33+ | 3 | 3 | 3 | | | | 53 | 4 | S | S | MSS |
| CN9934 | none | 3 | 3 | 3 | 3 | 33+ | 33+ | 3 | 3 | 3 | 3 | | | | 21 | 4 | R | R | MS |
| CN9936 | none | 3 | 3 | 3 | 3 | 33+ | 3 | 33+ | 3 | 3 | 3 | | | | 40 | 8 | R | MS | MS |
| CN12251 | none | 3 | 3 | 3 | 3 | 33+ | 33+ | 3 | 3 | 3 | 3 | | | | 13 | 6 | MRMS | R | MR |
| Dvina | none | 3 | 3 | 3 | 3 | 33+ | 33+ | 3 | 3 | 3 | 3 | | | | 70 | 6 | S | S | S |
| Golubka | none | 3 | 3 | 3 | 3 | 33+ | 33+ | 33+ | 3 | 3 | 3 | | | | 36 | 6 | ? | MS | MS |
| Gorkorskaja15 | none | 3 | 3 | 3 | 3 | 3 | 33+ | 33+ | 3 | 3 | 3 | | | | 25 | 3 | MRMS | MS | MS |
| Narinskajal | none | 3 | 3 | 3 | 3 | 33+ | 33+ | 33+ | 3 | 3 | 3 | | | | 66 | 5 | S | S | S |
| Odess kaja 13 | none | 3 | 3 | 3 | 3 | 3 | 33+ | 33+ | 3 | 3 | 3 | | | | 48 | 7 | S | S | S |
| Pamjat Urala | none | 3 | 3 | 3 | 3 | 33+ | 33+ | 33+ | 3 | 3 | 3 | | | | 65 | 4 | MSS | S | S |
| Severodvinskaja1 | none | 3 | 3 | 3 | 3 | 33+ | 33+ | 33+ | 3 | 3 | 3 | | | | 70 | 6 | S | S | S |
| Udarnica | none | 3 | 3 | 3 | 3 | 33+ | 33+ | 33+ | 3 | 3 | 3 | | | | 65 | 7 | S | S | S |
| CN11461 | none | 3 | 3 | 3 | 3 | 3 | 33+ | 33+ | 3 | 3 | 3 | | | | 41 | 4 | S | S | MSS |
| Comeback | none | 3 | 3 | 3 | 3 | 33+ | 33+ | 33+ | 3 | 3 | 3 | | | | 56 | 5 | MS | S | S |
| Fylby | none | 3 | 3 | 3 | 3 | 33- | 33+ | 33+ | 3 | 3 | 3 | | | | 57 | 4 | S | S | S |
| CN40750 | none | 3 | 3 | 3 | 3 | 33- | 33+ | 3 | 3 | 3 | 3 | | | | 50 | 3 | MRMS | MS | S |

| Accession name | Seedling genes | BBBD | MBDS | MGBJ | TJBJ | TDBG | MBRJ | PBDQ | THMJ | TNRJ | TCRJ | Marker data | | | MRS | SE | HR | | |
|---|---|---|---|---|---|---|---|---|---|---|---|---|---|---|---|---|---|---|---|
| | | | | | | | | | | | | *Lr1* | *Lr10* | *Lr34* | | | WPG | GLN | POR |
| Lada | none | 3 | 3 | 3 | 3 | 33- | 33+ | 3 | 3 | 3 | 3 | | | | 9 | 3 | MR | R | MS |
| Falerio | none | 3 | 3 | 3 | 3 | 33+ | 33+ | 3 | 3 | 3 | 3 | | | *Lr34* | 21 | 6 | MSS | R | R |
| Albimonte | none | 3 | 3 | 3 | 3 | 33+ | 33+ | 33+ | 3 | 3 | 3 | | | *Lr34* | 7 | 2 | MSS | R | MS |
| CN11642 | none | 3 | 3 | 3 | 3 | 33+ | 33+ | 3 | 3 | 3 | 3 | | | | 39 | 6 | S | S | MS |
| CN12361 | none | 3 | 3 | 3 | 3 | 33+ | 33+ | 33+ | 3 | 33+ | 3 | | | | 52 | 5 | MS | ? | ? |
| CN11720 | none | 3 | 3 | 3 | 3 | 33+ | 33+ | 3 | 3 | 3 | 3 | | | | 38 | 7 | MS | MS | MS |
| CN10661 | none | 3 | 3 | 3 | 3 | 33+ | 33+ | 3 | 3 | 3 | 3 | | | *Lr34* | 35 | 5 | ? | MS | MS |
| Bunyip | none | 3 | 3 | 3 | 3 | 33+ | 33+ | 3 | 3 | 3 | 3 | | | | 57 | 5 | S | S | MS |
| Bungulla | none | 3 | 3 | 3 | 3 | 33+ | 33+ | 3 | 3 | 3 | 3 | | | | 46 | 6 | MS | S | MS |
| Vijay | none | 3 | 3 | 3 | 3 | 33+ | 3 | 33+ | 3 | ? | 3 | | | | 24 | 4 | MS | S | R |
| Dorziyeh karak | none | 33+ | 3 | 33- | ? | 33+ | 3 | 3 | 3 | 3 | 3 | | | | 6 | 1 | ? | R | ? |
| Krasnaja Svenzda | none | 3 | 3 | 33- | 33+ | 33+ | 33+ | 33+ | 3 | 3 | 3 | | | | 54 | 5 | MS | S | S |
| Group2 | | | | | | | | | | | | | | | | | | | |
| CN99032 | *N* | 3 | 3 | 2 | 3 | 33+ | 3 | 33+ | 3 | 3 | 3 | | | | 1 | 0 | R | R | R |
| Preludio | *N* | 3 | 3 | 3 | 1 | 33+ | 33+ | 3 | 3 | 33+ | 3 | | | | 3 | 1 | MR | R | R |
| Kenya Farmer | *N* | 3 | 2 | 3 | 3 | 3 | 33+ | 3 | 3 | 3 | 3 | | | | 28 | 4 | MR | R | MS |

| Accession name | Seedling genes | BBBD | MBDS | MGBJ | TJBJ | TDBG | MBRJ | PBDQ | THMJ | TNRJ | TCRJ | Marker data | | | MRS | SE | HR | | |
|---|---|---|---|---|---|---|---|---|---|---|---|---|---|---|---|---|---|---|---|
| | | | | | | | | | | | | *Lr1* | *Lr10* | *Lr34* | | | WPG | GLN | POR |
| Santa Elena | *N* | 33+ | 3 | 3 | 3 | 21 | 3 | 3 | 3 | 3 | 3 | | | | 53 | 5 | MS | R | MS |
| CN9940 | *N* | 3 | 2 | 33- | 3 | 33+ | 3 | 33+ | 3 | 3 | 3 | | | | 10 | 2 | MRMS | MS | R |
| Beloglina | *N* | 3 | 3 | 3 | 3 | 33- | 3 | ;2 | 3 | 3 | 3 | | | | 34 | 4 | S | S | ? |
| StrubesFortschritt | *N* | 3 | 3 | 33- | 3 | 33+ | 33+ | ;1 | 3 | 3 | 3 | | | | 42 | 5 | MS | MS | MS |
| Atacatzo 1 | *N* | 3 | 3 | 3 | 33+ | 33+ | 33+ | ;1 | 3 | 3 | 3 | | | | 76 | 3 | S | S | S |
| CN10750 | *N* | 3 | 3 | 2 | 2 | 33+ | 33+ | 3 | 3 | 3 | 3 | | | | 44 | 6 | MSS | MSS | S |
| Klein Credito | *N* | 3 | 2 | 1 | 2 | 33+ | 3 | 33+ | 3 | 3 | 3 | | | | 17 | 3 | MR | MS | MS |
| CN12624 | *N* | 3 | 3 | 2 | 2 | 33+ | 2 | 3 | ;12 | 3 | 3 | | | | 7 | 2 | MR | R | R |
| Group 3 | | | | | | | | | | | | | | | | | | | |
| Subgroup 1 | | | | | | | | | | | | | | | | | | | |
| Red River 68 | *Lr1* | 0 | 3 | 3 | 1 | 33+ | 3 | 33+ | 3 | 33+ | 3 | *Lr1* | | | 13 | 3 | MR | MRMS | MS |
| Shu Chou Wheat #3 | *Lr1* | ; | 3 | 3 | 3 | 33+ | 33+ | 3 | 33+ | 3 | 3 | *Lr1* | | *Lr34* | 21 | 3 | MS | MSS | MSS |
| Shinchunaga | *Lr1* | 2 | 3 | 3 | 3 | 33+ | 33+ | 3 | 3 | 3 | 3 | *Lr1* | | | 37 | 5 | MSS | MSS | MS |
| Kostoff's Triple hybrid | *Lr1* | 1 | 3 | 3 | 3 | 3 | 33+ | 33+ | 3 | 3 | 3 | *Lr1* | | | 29 | 4 | MS | S | MS |
| Iskra | *Lr1* | 2 | 3 | 3 | 3 | 33+ | 33+ | 33+ | 3 | 3 | 3 | *Lr1* | | | 56 | 6 | R | MR | ? |

| Accession name | Seedling genes | BBBD | MBDS | MGBJ | TJBJ | TDBG | MBRJ | PBDQ | THMJ | TNRJ | TCRJ | Marker data | | | MRS | SE | HR | | |
|---|---|---|---|---|---|---|---|---|---|---|---|---|---|---|---|---|---|---|---|
| | | | | | | | | | | | | *Lr1* | *Lr10* | *Lr34* | | | WPG | GLN | POR |
| Fruher Tiroler Bin | *Lr1* | 2 | 3 | 3 | 3 | 3 | 33+ | 33+ | 3 | 3 | 3 | *Lr1* | | | 51 | 5 | MS | MSS | S |
| Ventura | *Lr1* | 1 | 3 | 3 | 3 | 33+ | 33+ | 3 | 3 | 3 | 3 | *Lr1* | | | 44 | 6 | MS | S | S |
| Endeavor | *Lr1* | 0 | 3 | 3 | 3 | 3 | 33+ | 3 | 3 | 3 | 3 | *Lr1* | | | 29 | 6 | MS | MS | S |
| cailloux | *Lr1* | 3 | 3 | 3 | 3 | 33+ | 33+ | 33+ | 3 | 33+ | 3 | *Lr1* | | | 55 | 3 | ? | S | S |
| CN12213 | *Lr1* | 3 | 3 | 3 | 3 | 33+ | 33+ | 33+ | 3 | 3 | 3 | *Lr1* | | | 65 | 4 | MSS | MSS | S |
| Shahpassand | *Lr1* | 3 | 3 | 3 | 3 | 33+ | 33+ | 33+ | 3 | 3 | 3 | *Lr1* | | | 84 | 2 | S | S | ? |
| Moskovka | *Lr1* | 3 | 3 | 3 | 3 | 33+ | 33+ | 33+ | 3 | 3 | 3 | *Lr1* | | *Lr34* | 53 | 5 | MS | MS | S |
| Buffum | *Lr1* | 3 | 3 | 3 | 3 | 33- | 33+ | 3 | 3 | 3 | 3 | *Lr1* | | | 48 | 4 | ? | S | S |
| Maja | *Lr1* | 3 | 3 | 3 | 3 | 33+ | 33+ | 3 | 3 | 3 | 3 | *Lr1* | | | 48 | 4 | MS | S | S |
| Kinsman | *Lr1* | 3 | 3 | 3 | 3 | 3+ | 33+ | 3 | 3 | 3 | 3 | *Lr1* | | | 8 | 3 | ? | S | S |
| Heines Peko | *Lr1* | 3 | 3 | 3 | 3 | 33+ | 33+ | 3 | 3 | 3 | 3 | *Lr1* | | | 32 | 6 | S | MSS | MSS |
| CN12425 | *Lr1* | 3 | 3 | 3 | 3 | 3 | 33+ | 3 | 3 | 3 | 3 | *Lr1* | | | 16 | 4 | MR | R | R |
| Ho chun No. 12 | *Lr10* | 2 | 3 | 3 | 3 | 33+ | 33+ | 33+ | 3 | 3 | 3 | | *Lr10* | | 48 | 5 | MS | S | MS |
| Etoile De Choisy | *Lr10* | 2 | 3 | 3 | 3 | 33+ | 33+ | 33+ | 3 | 3 | 3 | | *Lr10* | | 58 | 6 | S | MS | S |
| Magueija | *Lr10* | 2 | 3 | 3 | 3 | 33+ | 33+ | 3 | 3 | 3 | 3 | | *Lr10* | *Lr34* | 3 | 2 | R | ? | R |
| Bonza | *Lr10* | 0 | 3 | 3 | 3 | 33+ | 33+ | 3 | 3 | 3 | 3 | | *Lr10* | *Lr34* | 18 | 4 | MRMS | R | 5jw |

| Accession name | Seedling genes | BBBD | MBDS | MGBJ | TJBJ | TDBG | MBRJ | PBDQ | THMJ | TNRJ | TCRJ | Marker data | | | MRS | SE | HR | | |
|---|---|---|---|---|---|---|---|---|---|---|---|---|---|---|---|---|---|---|---|
| | | | | | | | | | | | | *Lr1* | *Lr10* | *Lr34* | | | WPG | GLN | POR |
| Chara | *Lr10* | 2 | 3 | 3 | 3 | 33+ | 33+ | 3 | 3 | 3 | 3 | | *Lr10* | *Lr34* | 30 | 6 | ? | S | ? |
| Federation | *Lr10* | 1 | 3 | 3 | 3 | 33+ | 33+ | 3 | 3 | 3 | 3 | | *Lr10* | | 60 | 6 | MS | S | MSS |
| CN33892 | *Lr10* | 2 | 3 | 3 | 3 | 33+ | ? | 33+ | 3 | 3 | 3 | | *Lr10* | | 25 | 5 | S | S | R |
| CN33893 | *Lr10* | 3 | 3 | 3 | 3 | 33+ | 33+ | 33+ | 3 | 3 | 3 | | *Lr10* | | 7 | 3 | R | R | R |
| Kenphad 25 | *Lr10* | 3 | 3 | 3 | 3 | 33+ | 3 | 33+ | 3 | 3 | 3 | | *Lr10* | | 46 | 4 | MRMS | MSS | MSS |
| Sonalika | *Lr10* | 33+ | 3 | 3 | 3 | 3 | 33+ | 33+ | 3 | 3 | 3 | | *Lr10* | | 47 | 6 | MS | S | R |
| Irkutskaja 49 | *Lr10* | 3 | 3 | 3 | 3 | 33+ | 33+ | 33+ | 3 | 3 | 3 | | *Lr10* | | 67 | 6 | S | S | S |
| Krasnokutka 3 | *Lr10* | 3 | 3 | 3 | 3 | 33+ | 33+ | 3 | 3 | 3 | 3 | | *Lr10* | | 72 | 4 | MSS | S | S |
| CN9794 | *Lr10* | 3 | 3 | 3 | 3 | 33+ | 3 | 33+ | 3 | 3 | 3 | | *Lr10* | | 25 | 6 | ? | ? | R |
| Hard Federation | *Lr10* | 3 | 3 | 3 | 3 | 33+ | 33+ | 3 | 3 | 3 | 3 | | *Lr10* | | 50 | 5 | S | S | MS |
| Hofed | *Lr10* | 3 | 3 | 3 | 3 | 33+ | 3 | 3 | 3 | 3 | 3 | | *Lr10* | | 44 | 4 | MRMS | S | S |
| Sinvalocho | *Lr3* | 0 | 3 | 3 | 3 | 33+ | 33+ | 3 | 3 | 33+ | 3 | | | *Lr34* | 18 | 3 | S | MS | R |
| CN2758 | *Lr3* | 1 | 3 | 3 | 3 | 33+ | 33+ | 33+ | 3 | 33+ | 33+ | | | | 7 | 2 | MS | MS | MS |
| CN11647 | *Lr3* | 2 | 3 | 3 | 3 | 33+ | 33+ | 33+ | 3 | 3 | 3 | | | | 43 | 6 | S | S | S |
| CN33803 | *Lr3* | 2 | 3 | 3 | 3 | 33+ | 33+ | 33+ | 3 | 3 | 3 | | | | 17 | 5 | MS | R | R |
| Su Mai No 3 | *Lr3* | 1 | 3 | 3 | 3 | 33+ | 33+ | 3 | 3 | 3 | 3 | | | *Lr34* | 47 | 7 | S | S | S |

| Accession name | Seedling genes | BBBD | MBDS | MGBJ | TJBJ | TDBG | MBRJ | PBDQ | THMJ | TNRJ | TCRJ | Marker data | | | MRS | SE | HR | | |
|---|---|---|---|---|---|---|---|---|---|---|---|---|---|---|---|---|---|---|---|
| | | | | | | | | | | | | *Lr1* | *Lr10* | *Lr34* | | | WPG | GLN | POR |
| Azar | *Lr3* | 2 | 3 | 3 | 3 | 33+ | 33+ | 3 | 3 | 3 | 3 | | | | 73 | 3 | S | S | S |
| Norin 75 | *Lr3* | 2 | 3 | 3 | 3 | 33+ | 3 | 4 | 3 | 3 | 3 | | | | 71 | 4 | ? | ? | S |
| Nobeoka Bozu | *Lr3* | 2 | 3 | 3 | 3 | 33+ | 33+ | 33+ | 3 | 3 | 3 | | | *Lr34* | 14 | 2 | MS | MS | S |
| Ikuzai #1 | *Lr3* | 2 | 3 | 3 | 3 | 33+ | 33+ | 33+ | 3 | 3 | 3 | | | *Lr34* | 42 | 7 | ? | S | ? |
| CN42522 | *Lr3* | 2 | 3 | 3 | 3 | 33+ | 33+ | 3 | 3 | 3 | 3 | | | | 70 | 3 | MS | R | MS |
| CN29871 | *Lr3* | 2 | 3 | 3 | 3 | 33+ | 33+ | 33+ | 3 | 3 | 3 | | | | 61 | 5 | MS | R | MS |
| CN29858 | *Lr3* | 2 | 3 | 3 | 3 | 33+ | 33+ | 33+ | 3 | 3 | 3 | | | | 69 | 2 | S | S | S |
| CN10241 | *Lr3* | 2 | 3 | 3 | 3 | 33+ | 33+ | 3 | 3 | 3 | 3 | | | | 15 | 4 | ? | R | ? |
| Ferrugineum87 | *Lr3* | 2 | 3 | 3 | 3 | 33+ | 33+ | 33+ | 3 | 3 | 3 | | | | 18 | 3 | MS | MS | MS |
| karagandinskaja | *Lr3* | 2 | 3 | 3 | 3 | 33+ | 33+ | 33+ | 3 | 3 | 3 | | | | 44 | 6 | MS | MS | MSS |
| Vega | *Lr3* | 2 | 3 | 3 | 3 | 33+ | 33+ | 33+ | 3 | 3 | 3 | | | | 46 | 5 | ? | S | MSS |
| 71GN No 115 | *Lr3* | 2 | 3 | 3 | 3 | 3 | 33+ | 33+ | 3 | 3 | 3 | | | | 51 | 7 | MR | MS | MS |
| Sandra | *Lr3* | 1 | 3 | 3 | 3 | 33+ | 33+ | 3 | 3 | 3 | 3 | | | | 51 | 4 | MS | S | S |
| CN32504 | *Lr3* | 2 | 3 | 3 | 3 | 33+ | 33+ | 3 | 3 | 3 | 3 | | | *Lr34* | 31 | 6 | S | RMR | R |
| Argentina | *Lr3* | 1 | 3 | 3 | 3 | 33+ | 33+ | 3 | 3 | 3 | 3 | | | *Lr34* | 17 | 5 | MS | R | R |
| Klein otto Wulff | *Lr3* | 0 | 3 | 3 | 3 | 33+ | 33+ | 3 | 3 | 3 | 3 | | | | 17 | 4 | MS | MSS | MS |

| Accession name | Seedling genes | BBBD | MBDS | MGBJ | TJBJ | TDBG | MBRJ | PBDQ | THMJ | TNRJ | TCRJ | Marker data | | | MRS | SE | HR | | |
|---|---|---|---|---|---|---|---|---|---|---|---|---|---|---|---|---|---|---|---|
| | | | | | | | | | | | | *Lr1* | *Lr10* | *Lr34* | | | WPG | GLN | POR |
| CN11307 | *Lr3* | ; | 3 | 3 | 3 | 33+ | 33+ | 3 | 3 | 3 | 3 | | | | 52 | 6 | S | S | MS |
| Gabo | *Lr3* | 1 | 3 | 3 | 3 | 33+ | 33+ | 3 | 3 | 3 | 3 | | | | 59 | 3 | MS | S | S |
| Hilgendorf | *Lr3* | 1 | 3 | 3 | 3 | 33+ | 3 | 3 | 3 | 3 | 3 | | | *Lr34* | 1 | 0 | MS | R | R |
| Sunbird | *Lr9* | 0 | ; | ; | 0 | 23 | 0 | x | ; | 3 | ; | | | *Lr34* | 35 | 6 | MS | R | MS |
| Onohoiskaja 4 | *Lr20* | 3 | 3 | 3 | 3 | 22+ | 33+ | 33+ | 3 | 3 | 3 | | | | 45 | 4 | MS | MS | S |
| Ladoga | *Lr20* | 3 | 3 | 3 | 3 | X | 33+ | 33+ | 3 | 3 | 3 | | | | 55 | 5 | S | S | S |
| Janetzkis Probat | *Lr20* | 3 | 3 | 3 | 3 | 22+ | 33+ | 3 | 3 | 3 | 3 | | | | 41 | 5 | MSS | S | S |
| Da Maia | *Lr20* | 3 | 3 | 3 | 3 | 22+ | 33+ | 3 | 3 | 3 | 3 | | | | 41 | 4 | MS | MSS | MS |
| Red Marvel | *Lr20* | 3 | 3 | 3 | 3 | 22+ | 33+ | 33+ | 3 | 3 | 3 | | | | 12 | 4 | MS | R | ? |
| Little Joss | *Lr20* | 3 | 3 | 3 | 3 | 22+ | 33+ | 3 | 3 | 3 | 3 | | | | 46 | 6 | MS | MS | MSS |
| Holdfast | *Lr20* | 3 | 3 | 3 | 3 | 22+ | 33+ | 3 | 3 | 3 | 3 | | | | 1 | 0 | MS | R | MS |
| CN6030 | *Lr20* | 3 | 3 | 3 | 3 | 22+ | 33+ | 3 | 3 | 3 | 3 | | | *Lr34* | 41 | 7 | MR | S | MS |
| John Brown | *Lr20* | 33+ | 3 | 3 | 3 | 2 | 33+ | 3 | 3 | 3 | 3 | | | *Lr34* | 43 | 5 | MRMS | S | S |
| Cross 7 | *Lr20* | 3 | 3 | 3 | 3 | ; | 33+ | 3 | 3 | 3 | 3 | | | | 39 | 7 | MS | MS | S |
| Oroua | *Lr20* | 3 | 3 | 3 | 3 | 21 | 33+ | 3 | 3 | 3 | 3 | | | | 14 | 2 | MR | MS | MS |
| Giza 141 | *Lr20* | 3 | 3 | 3 | ? | 22+ | 33+ | 33+ | 3 | 3 | 3 | | | | 18 | 5 | ? | MSS | R |

| Accession name | Seedling genes | BBBD | MBDS | MGBJ | TJBJ | TDBG | MBRJ | PBDQ | THMJ | TNRJ | TCRJ | Marker data | | | MRS | SE | HR | | |
|---|---|---|---|---|---|---|---|---|---|---|---|---|---|---|---|---|---|---|---|
| | | | | | | | | | | | | *Lr1* | *Lr10* | *Lr34* | | | WPG | GLN | POR |
| Fronteira | *Lr20* | 3 | 3 | 3 | 33+ | 22+ | 33+ | 3 | 3 | 3 | 3 | | | | 25 | 3 | MR | S | S |
| Janetzkis FruherS | *Lr28* | 2 | 2 | 3 | 3 | 33+ | 33+ | 33+ | 3 | 3 | 3 | | | | 45 | 4 | S | S | S |
| Prospur | *Lr28* | 0 | 2 | 3 | 3 | 33- | 33+ | 33+ | 3 | 3 | 3 | | | *Lr34* | 36 | 4 | MR | MSS | MS |
| Subgroup 2 | | | | | | | | | | | | | | | | | | | |
| Glenavon | *Lr1,Lr10* | 0 | 3 | 3 | 3 | 33+ | 33+ | 3 | 3 | 33+ | 3 | *Lr1* | *Lr10* | *Lr34* | 28 | 5 | RMR | ? | MS |
| Koktunkulskaja 332 | *Lr1,Lr10* | 0 | 3 | 3 | 3 | 33+ | 33+ | 33+ | 3 | 3 | 3 | *Lr1* | *Lr10* | | 47 | 4 | MSS | S | MS |
| Cargimarec | *Lr1,Lr10* | 0 | 3 | 3 | 3 | 33+ | 33+ | 3 | 3 | 3 | 3 | *Lr1* | *Lr10* | | 22 | 5 | ? | S | S |
| Hohenhein | *Lr1,Lr10* | ; | 3 | 3 | 3 | 3 | 33+ | 3 | 3 | 3 | 3 | *Lr1* | *Lr10* | | 50 | 6 | S | S | MSS |
| Jakutjanka | *Lr1,Lr10* | 3 | 3 | 3 | 3 | 3 | 33+ | 33+ | 3 | 3 | 3 | *Lr1* | *Lr10* | | 65 | 4 | 80 | S | S |
| Kamalinka | *Lr1,Lr10* | 3 | 3 | 3 | 3 | 33+ | 33+ | 33+ | 3 | 3 | 3 | *Lr1* | *Lr10* | | 49 | 5 | MS | ? | MS |
| Krasnojarskaja 1103 | *Lr1,Lr10* | 3 | 3 | 3 | 3 | 33+ | 33+ | 3 | 3 | 3 | 3 | *Lr1* | *Lr10* | | 69 | 3 | S | S | S |
| Penkop | *Lr1,Lr10* | 3 | 3 | 3 | 3 | 33+ | 33+ | 3 | 3 | 3 | 3 | *Lr1* | *Lr10* | | 64 | 4 | MSS | S | MSS |
| White Federation | *Lr1,Lr10* | 3 | 3 | 3 | 3 | ? | 33+ | 3 | 3 | 3 | 3 | *Lr1* | *Lr10* | | 59 | 6 | ? | S | R |
| Carina | *Lr1,Lr2c* | 0 | 0 | 0 | 3 | 33+ | 0 | 33+ | 3 | 3 | 3 | *Lr1* | | | 65 | 4 | S | MSS | S |
| Hsin ShuKuan#1 | *Lr1,Lr14a* | 3 | 3 | 3 | 3 | X | 33+ | x | 3 | 33+ | 3 | *Lr1* | | | 50 | 5 | MSS | S | MS |

| Accession name | Seedling genes | BBBD | MBDS | MGBJ | TJBJ | TDBG | MBRJ | PBDQ | THMJ | TNRJ | TCRJ | Marker data | | | MRS | SE | HR | | |
|---|---|---|---|---|---|---|---|---|---|---|---|---|---|---|---|---|---|---|---|
| | | | | | | | | | | | | *Lr1* | *Lr10* | *Lr34* | | | WPG | GLN | POR |
| New Pusa | *Lr1,Lr20* | 2 | 3 | 3 | 3 | ; | 33+ | 3 | 3 | 3 | 3 | *Lr1* | | | 25 | 4 | MR | MS | MS |
| Retacon INTA | *Lr1,Lr20* | 0 | 3 | 3 | 2 | 33+ | 33+ | 33+ | 3 | 3 | 3 | *Lr1* | | | 22 | 5 | MS | S | MS |
| CN12666 | *Lr1,Lr20* | 1 | 3 | 3 | 3 | 0 | 33+ | 33+ | 3 | 3 | 3 | *Lr1* | | *Lr34* | 46 | 4 | ? | S | MS |
| CN6025 | *Lr1,Lr20* | 0 | 3 | 3 | 3 | 22+ | 33+ | 3 | 3 | 3 | 3 | *Lr1* | | | 58 | 5 | S | S | S |
| Sabre | *Lr1,Lr20* | 1 | 3 | 3 | 3 | 2 | 3 | 3 | 3 | 3 | 3 | *Lr1* | | *Lr34* | 34 | 7 | S | ? | MS |
| Pobeda | *Lr1,Lr20* | 3 | 3 | 3 | 3 | 2 | 33+ | 33+ | 3 | 3 | 3 | *Lr1* | | | 66 | 4 | MSS | S | S |
| CN12442 | *Lr1,Lr20* | 3 | 3 | 3 | 3 | 22+ | 33+ | 3 | 3 | 3 | 3 | *Lr1* | | | 47 | 6 | MS | S | S |
| Kenya Governor | *Lr1,Lr20* | 3 | 3 | 3 | 3 | 22+ | 3 | 3 | 3 | 3 | 3 | *Lr1* | | *Lr34* | 30 | 4 | MS | S | MSS |
| NING 8331 | *Lr1,Lr26* | 1 | 2 | 1 | ; | X | ; | ; | 3 | ; | 33- | *Lr1* | | | 3 | 1 | ? | ? | ? |
| Wildcat | *Lr1,N* | 0 | 3 | 3 | 3 | 33+ | 33+ | X | 3 | 33+ | 3 | *Lr1* | | | 66 | 5 | MRMS | MSS | S |
| Xiang Mai No 1 | *Lr1,N* | 1 | 3 | 3 | 2 | 33+ | 3 | 3 | 3 | 3 | 3 | *Lr1* | | | 9 | 3 | MR | MS | R |
| Touse | *Lr1,N* | 2 | 3 | 3 | 0 | 33+ | 33+ | 33+ | 3 | 3 | 3 | *Lr1* | | | 69 | 3 | S | S | MS |
| Buck Pucara | *Lr1,N* | 0 | 3 | 3 | 3 | 33+ | 33+ | ;1 | 3 | 3 | 3 | *Lr1* | | | 19 | 4 | MR | R | MS |
| Kukri | *Lr1,N* | 0 | 3 | 3 | 2 | 33- | 33+ | 3 | 3 | 3 | 3 | *Lr1* | | | 26 | 5 | MRMS | MS | MSS |
| Tobari F66 | *Lr1,N* | 0 | 3 | 3 | 2 | 33- | 33+ | ; | 3 | 3 | 3 | *Lr1* | | | 26 | 5 | MRMS | MS | R |
| CDC Teal | *Lr1,N* | 2 | 3 | 2 | 1 | 33+ | X | 0 | 3 | 21 | 3 | *Lr1* | | *Lr34* | 16 | 4 | MR | MS | MS |

| Accession name | Seedling genes | BBBD | MBDS | MGBJ | TJBJ | TDBG | MBRJ | PBDQ | THMJ | TNRJ | TCRJ | Marker data | | | MRS | SE | HR | | |
|---|---|---|---|---|---|---|---|---|---|---|---|---|---|---|---|---|---|---|---|
| | | | | | | | | | | | | *Lr1* | *Lr10* | *Lr34* | | | WPG | GLN | POR |
| Klein Universal | *Lr1,N* | 3 | 3 | 0 | 2 | 33- | 3 | 3 | 3 | 3 | 3 | *Lr1* | | | 10 | 4 | MS | R | MSS |
| Fengcheung No.1 | *Lr1,N* | 3 | 3 | 2 | 3 | 33+ | 3 | 33+ | 3 | 3 | 3 | *Lr1* | | | 78 | 2 | S | S | S |
| Jufy 2 | *Lr1,N* | 3 | 3 | 3 | 2 | 33+ | 33+ | 3 | 3 | 3 | 3 | *Lr1* | | | 65 | 4 | SMS | S | S |
| Bersee | *Lr10,Lr14a* | 3 | 3 | 3 | 3 | 33+ | 33+ | 3 | 3 | 3 | 3 | | *Lr10* | | 15 | 3 | MR | RMR | R |
| Pirana | *Lr10,Lr20* | 2 | 3 | 3 | 3 | ; | 33+ | 3 | 3 | 3 | 3 | | *Lr10* | *Lr34* | 15 | 3 | MS | S | MS |
| Dominator | *Lr10,Lr20* | 1 | 3 | 3 | 3 | 22+ | 33+ | 33+ | 3 | 3 | 3 | | *Lr10* | | 70 | 4 | SMS | S | S |
| CN6024 | *Lr10,Lr20* | 1 | 3 | 3 | 3 | ;12 | 33+ | 3 | 3 | 3 | 3 | | *Lr10* | | 41 | 4 | S | S | S |
| CN12388 | *Lr10,Lr20* | 2 | 3 | 3 | 3 | 22+ | 33+ | 3 | 3 | 3 | 3 | | *Lr10* | *Lr34* | 27 | 3 | MS | MS | MS |
| Daeraad | *Lr10,Lr20* | 1 | 3 | 3 | 3 | 0 | 3 | 3 | 3 | 3 | 33- | | *Lr10* | *Lr34* | 36 | 5 | MRMS | S | S |
| CD87 | *Lr10,Lr20* | ; | 3 | 3 | 3 | 2 | 33+ | 3 | 3 | 3 | 3 | | *Lr10* | | 36 | 4 | MS | S | S |
| CN12035 | *Lr10,Lr20* | 1 | 3 | 3 | 3 | 0 | 33+ | 3 | 3 | 3 | 3 | | *Lr10* | | 47 | 7 | MR | R | R |
| Trintani | *Lr10,Lr20* | 3 | 3 | 3 | 3 | 22+ | 33+ | 33+ | 3 | 3 | 3 | | *Lr10* | | 37 | 6 | MRMS | S | S |
| Olaeta Calandria | *Lr10,Lr20* | 3 | 3 | 3 | 3 | X | 33+ | 33+ | 3 | 33+ | 3 | | *Lr10* | | 69 | 4 | MS | S | MS |
| CN1849 | *Lr10,Lr28* | 2 | 1 | 3 | 3 | 33+ | 33+ | 3 | 3 | 3 | 3 | | *Lr10* | | 47 | 4 | MS | MSS | MS |
| CN12261 | *Lr10,N* | 1 | 3 | 1 | 3 | 33+ | 22+ | 33+ | 3 | 33+ | 33- | | *Lr10* | | 20 | 3 | MRMS | R | MS |
| Hybride DuJubile | *Lr10,N* | 3 | 3 | 3 | 3 | 33- | 22+ | 3 | 3 | 3 | 3 | | *Lr10* | | 56 | 3 | S | S | MSS |

| Accession name | Seedling genes | BBBD | MBDS | MGBJ | TJBJ | TDBG | MBRJ | PBDQ | THMJ | TNRJ | TCRJ | Marker data | | | MRS | SE | HR | | |
|---|---|---|---|---|---|---|---|---|---|---|---|---|---|---|---|---|---|---|---|
| | | | | | | | | | | | | *Lr1* | *Lr10* | *Lr34* | | | WPG | GLN | POR |
| Phoebus | *Lr10,N* | 3 | 3 | 1 | 3 | 33- | 33+ | 3 | 3 | 3+ | 33-+ | | *Lr10* | | 9 | 4 | MR | R | R |
| Werna | *Lr10,N* | 3 | 2 | 3 | 3 | 33+ | 2 | 33+ | 3 | 3 | 3 | | *Lr10* | | 31 | 3 | MR | R | S |
| Combate | *Lr10,N* | 3 | 3 | 3 | 3 | 33+ | 3 | x | 3 | 3 | 33+ | | *Lr10* | | 22 | 3 | MR | S | S |
| Arawa | *Lr3,Lr14a* | 2 | 3 | 3 | 3 | 21 | 33+ | ;1 | 3 | 3 | 3 | | | | 13 | 2 | MRMS | R | R |
| Ladoga | *Lr3,Lr15* | 0 | 3 | 3 | 3 | 33+ | 33+ | 33+ | 3 | ;12 | 3 | | | | 71 | 5 | MS | MS | S |
| KVL 2263 | *Lr3,Lr20* | 2 | 3 | 3 | 3 | ; | 33+ | 3 | 3 | 3 | 3 | | | *Lr34* | 8 | 1 | R | R | MRMS |
| Chengdu Guangtou | *Lr3,Lr20* | 1 | 3 | 3 | 3 | ; | 3 | 3 | 3 | 3 | 3 | | | | 6 | 2 | MRMS | R | S |
| Nan Tong DA Huang PI | *Lr3,Lr20* | 0 | 3 | 3 | 3 | ;1 | 33+ | 3 | 3 | 3 | 3 | | | | 13 | 3 | MR | MS | ? |
| Minskaja | *Lr3,Lr20* | ; | 3 | 3 | 3 | ; | 33+ | 33+ | 3 | 3 | 3 | | | | 50 | 4 | S | S | MSS |
| Setter | *Lr3,Lr20* | 2 | 3 | 3 | 3 | 0 | 33+ | 3 | 3 | 3 | 3 | | | | 18 | 4 | S | R | MS |
| Musket | *Lr3,Lr20* | 2 | 3 | 3 | 3 | 22+ | 33+ | 3 | 3 | 3 | 3 | | | | 24 | 5 | MS | MSS | MS |
| Tonic | *Lr3,Lr20* | 2 | 3 | 3 | 3 | 0 | 33+ | 3 | 3 | 3 | 3 | | | | 34 | 5 | MRMS | MS | MS |
| Hope | *Lr3,Lr20* | 0 | 3 | 3 | 3 | ;12 | 33+ | 3 | 3 | 3 | 3 | | | *Lr34* | 52 | 4 | MRMS | MSS | MSS |
| Klein Toledo | *Lr3,Lr20* | 2 | 3 | 3 | 3 | 1 | 33+ | 3 | 3 | 3 | 3 | | | | 4 | 1 | R | R | R |
| Calidad | *Lr3,Lr20* | 0 | 3 | 3 | 3 | X | 33+ | 33+ | 3 | 3 | 3 | | | | 30 | 6 | MS | MS | MS |

| Accession name | Seedling genes | BBBD | MBDS | MGBJ | TJBJ | TDBG | MBRJ | PBDQ | THMJ | TNRJ | TCRJ | Marker data | | | MRS | SE | HR | | |
|---|---|---|---|---|---|---|---|---|---|---|---|---|---|---|---|---|---|---|---|
| | | | | | | | | | | | | *Lr1* | *Lr10* | *Lr34* | | | WPG | GLN | POR |
| Surpresa | *Lr3,Lr20* | 1 | 3 | 3 | 3 | 22+ | 33+ | 3- | 3 | 3 | 3 | | | | 25 | 4 | MR | S | MSS |
| Toropi | *Lr3,Lr20* | 2 | 3 | 3 | 3 | 22+ | 33+ | 3 | 3 | 3 | 3 | | | | 8 | 2 | ? | MS | MS |
| Menkemen | *Lr3,Lr20* | 0 | 3 | 3 | 3 | 22+ | 3 | 3 | 3 | 3 | 3 | | | *Lr34* | 22 | 4 | ? | MSS | R |
| Maribal 50 | *Lr3,Lr20* | ; | 3 | 3 | 3 | 22+ | 33+ | 3 | 3 | 3 | 3 | | | | 38 | 5 | MS | S | S |
| Sunstar | *Lr3,N* | 2 | 3 | 3 | 3 | 3 | 33+ | 3 | 3 | 3 | 0 | | | | 37 | 6 | MS | MS | MS |
| Prinqual | *Lr3,N* | ; | 3 | 0 | 3 | 33+ | 33+ | 3 | 3 | 3 | 3 | | | *Lr34* | 25 | 3 | MR | MS | MS |
| CN12268 | *Lr3,N* | 2 | 3 | 3 | 3 | 33+ | 33+ | ; | 3 | 3 | 3 | | | | 31 | 4 | R | S | MS |
| Rayhany | *Lr3,N* | `1 | 3 | 2 | 3 | 33+ | ? | 33+ | 3 | 3 | 3 | | | | 76 | 6 | S | S | S |
| Froubou | *Lr3,N* | 1 | 3 | 3 | 1 | 33+ | 33+ | 3 | 3 | 3 | 3 | | | | 2 | 1 | R | R | R |
| Gogatsukomugai | *Lr3,N* | 2 | 3 | 2 | 3 | 33+ | 33+ | 33+ | 3 | 3 | 3 | | | | 39 | 6 | MS | S | MSS |
| HY 320 | *Lr3,N* | 2 | 3 | 3 | 2 | 33+ | 3 | 3- | 3 | 33+ | 0 | | | | 39 | 5 | R | MRMS | ? |
| Funo | *Lr3,N* | 2 | 3 | 3 | 3 | 33+ | 22+ | 3 | 3 | 33+ | 2 | | | | 38 | 4 | MS | MS | MS |
| Squarhead's Master | *Lr3,N* | 2 | 3 | 2 | 3 | 33+ | 33+ | x | 3 | 3 | 3 | | | | 60 | 5 | S | ? | S |
| Bola Picota | *Lr3,N* | 1 | 3 | 3 | 3 | 33+ | 2 | ; | 3 | 3 | 3 | | | | 31 | 5 | MS | R | MS |
| El Gaucho | *Lr2c,N* | 2 | 1 | 0 | ; | 3 | 1 | 3 | 3 | 3 | 3 | | | *Lr34* | 2 | 1 | MRMS | R | R |

| Accession name | Seedling genes | BBBD | MBDS | MGBJ | TJBJ | TDBG | MBRJ | PBDQ | THMJ | TNRJ | TCRJ | Marker data | | | MRS | SE | HR | | |
|---|---|---|---|---|---|---|---|---|---|---|---|---|---|---|---|---|---|---|---|
| | | | | | | | | | | | | *Lr1* | *Lr10* | *Lr34* | | | WPG | GLN | POR |
| Tropeano | *Lr14a,N* | 3 | 2 | 3 | 2 | 22+ | 33+ | x | 3 | 3 | 3 | | | *Lr34* | 5 | 2 | R | MS | R |
| NING 8026 | *Lr15,N* | 2 | 3 | 12 | 2 | 33+ | ; | ;12 | 3 | ;1 | 3 | | | | 52 | 4 | MRMS | MS | MS |
| CN11698 | *Lr15,N* | 3 | 3 | 2 | 2 | 3 | 1 | 3 | ; | ; | ; | | | | 10 | 2 | MR | R | R |
| Warden | *Lr20,Lr28* | 2 | 1 | 3 | 3 | 0 | 33+ | 33+ | 3 | 33+ | 3 | | | | 45 | 5 | MS | S | MS |
| DART | *Lr20,Lr28* | 3 | 2 | 3 | 3 | 21 | 33+ | 3 | 3 | 3 | 3 | | | | 54 | 6 | MS | S | S |
| BAART | *Lr20,Lr28* | 1 | 2 | 3 | 3 | 22+ | 33+ | 3 | 33+ | 3 | 3 | | | | 51 | 5 | S | S | MS |
| Girija | *Lr20,N* | 3 | 3 | 3 | 2 | 0 | 3- | 3 | 3 | 3 | 3 | | | *Lr34* | 11 | 3 | MS | R | R |
| Kozlodui | *Lr20,N* | 3 | 2 | 3 | 3 | 2; | 33+ | 3 | 3 | 3 | 3 | | | | 61 | 7 | S | S | S |
| Acadimia 48 | *Lr20,N* | 3 | 3 | 3 | 3 | 22+ | 22+ | 3 | 3 | 3 | 3 | | | | 37 | 5 | MS | MSS | MS |
| Mentana | *Lr20,N* | 3 | 2 | 3 | 3 | 22+ | 33+ | 3 | 3 | 3 | 3 | | | *Lr34* | 36 | 5 | MS | S | S |
| Equator | *Lr20,N* | 3 | 2 | 3 | 3 | 22+ | 3 | 3 | 3 | 3 | 3 | | | | 30 | 7 | S | S | S |
| Kenya 58 | *Lr20,N* | 3 | 3 | 3 | 2 | ;12 | 33+ | 3 | 3 | 3 | 3 | | | *Lr34* | 17 | 3 | MS | MS | MS |
| Pacific Bluestem | *Lr20,N* | 33+ | 2 | 3 | 3 | 22+ | 3 | 3 | 3 | 3 | 3 | | | | 35 | 5 | MS | R | MS |
| CN30213 | *Lr20,N* | 3 | 2 | 2 | 3 | ; | 3 | 3 | 3 | 3 | 3 | | | | 31 | 4 | MRMS | MSS | MS |
| Benvenuto 3085 | *Lr20,N* | 3 | 2 | 3 | 2 | X | 33+ | 33+ | 3 | 3 | 3 | | | *Lr34* | 4 | 1 | MS | R | R |
| Colonias | *Lr20,N* | 1 | 3 | 2 | 2 | 21 | 33+ | 3 | 3 | 33+ | 3 | | | | 16 | 3 | MRMS | S | R |

| Accession name | Seedling genes | BBBD | MBDS | MGBJ | TJBJ | TDBG | MBRJ | PBDQ | THMJ | TNRJ | TCRJ | Marker data | | | MRS | SE | HR | | |
|---|---|---|---|---|---|---|---|---|---|---|---|---|---|---|---|---|---|---|---|
| | | | | | | | | | | | | *Lr1* | *Lr10* | *Lr34* | | | WPG | GLN | POR |
| Camadi | *Lr20,N* | 3 | 1 | ; | 2 | 22+ | 2 | 3 | 3- | 3 | 3 | | | | 4 | 1 | R | MR | R |
| Encruzilhada | *Lr28,N* | 2 | 0 | 2 | 2 | 33+ | 33+ | 33+ | 3 | 3 | 3 | | | | 6 | 2 | MR | R | R |
| Arcola | *Lr28,N* | 2 | 1 | 2 | 3 | 33+ | 3 | 3 | 33+ | 3 | 3 | | | | 3 | 1 | R | ? | MS |
| Lom | *Lr28,N* | 2 | 1 | 1 | 3 | 3 | 33+ | 3 | 3 | 3+ | 3 | | | | 17 | 5 | R | R | R |
| Dorziyeh Safra | *Lr28,N* | 1 | 1 | 0 | 2 | 33+ | 3 | 3 | 3 | 3 | 3 | | | | 5 | 1 | R | MS | ? |
| Pampa INTA | *Lr28,N* | 2 | 2 | 2 | 3 | 2 | 33+ | 3 | 3 | 3 | 3 | | | *Lr34* | 3 | 1 | MR | R | MS |
| AC Minto | *Lr28,N* | 1 | 2 | 1 | 2 | 33+ | 33+ | ;3 | 3 | 3 | 3 | | | | 8 | 2 | R | RMR | R |
| Subgroup 3 | | | | | | | | | | | | | | | | | | | |
| Jumbuck | *Lr3,Lr20,N* | 1 | 3 | 3 | 2 | ; | 33+ | 3 | 3 | 3 | 3 | | | *Lr34* | 48 | 5 | MRMS | S | MS |
| Bluesky | *Lr1,Lr10,N* | 0 | 3 | 2 | 3 | 33+ | 33+ | 33+ | 3 | 3 | 3 | *Lr1* | *Lr10* | | 19 | 3 | MS | MS | MR |
| RL4452 | *Lr1,Lr10,N* | 0 | 3 | 2 | 3 | 33+ | 3 | 3 | 3 | 33+ | 3 | *Lr1* | *Lr10* | | 28 | 5 | MRMS | MS | S |
| Roblin | *Lr1,Lr10,N* | 0 | 3 | 3 | 2 | 33+ | 33+ | 3 | 3 | 3 | 3 | *Lr1* | *Lr10* | *Lr34* | 30 | 5 | MR | SMS | MS |
| Corrine | *Lr1,Lr10,N* | 0 | 3 | 2 | 3 | 33+ | 3 | 3 | 3 | 3 | 3 | *Lr1* | *Lr10* | | 16 | 2 | R | SMS | MS |
| Laura | *Lr1,Lr10,N* | 0 | 3 | 2 | 3 | 33+ | 33+ | 3 | 3 | 3 | 3 | *Lr1* | *Lr10* | *Lr34* | 5 | 2 | MR | R | R |
| BW 90 | *Lr1,Lr10,N* | 2 | 3 | 3 | 12 | 33+ | 3 | 3 | 3 | 3 | 3 | *Lr1* | *Lr10* | | 13 | 2 | MRMS | MS | MS |
| Otane | *Lr1,Lr10,Lr20* | ; | 3 | 3 | 3 | 2 | 3 | 33+ | 3 | 3 | 3 | *Lr1* | *Lr10* | | 31 | 5 | MRMS | S | MS |

| Accession name | Seedling genes | BBBD | MBDS | MGBJ | TJBJ | TDBG | MBRJ | PBDQ | THMJ | TNRJ | TCRJ | Marker data | | | MRS | SE | HR | | |
|---|---|---|---|---|---|---|---|---|---|---|---|---|---|---|---|---|---|---|---|
| | | | | | | | | | | | | *Lr1* | *Lr10* | *Lr34* | | | WPG | GLN | POR |
| Biggar | *Lr3,15,N* | 2 | 3 | 3 | 2 | 33+ | 3 | 3 | 3 | 22+ | 3 | | | | 28 | 3 | ? | R | S |
| CN11204 | *Lr1,Lr20,N* | 2 | 3 | 3 | 3 | ; | 22+ | 33+ | 3 | 3 | 3 | *Lr1* | | *Lr34* | 8 | 2 | MS | MS | MS |
| Katja A1 | *Lr1,Lr20,Lr28* | ; | 2 | 3 | 3 | 32 | 33+ | 33+ | 3 | 3 | 3 | *Lr1* | | | 24 | 6 | S | S | MS |
| Parana | *Lr10,Lr20,N* | ; | 3 | 3 | 2 | 22+ | 3 | 3 | 3 | 3 | 3 | | *Lr10* | | 19 | 4 | MS | R | R |
| CN6171 | *Lr3,Lr20,N* | 1 | 3 | 3 | 2 | 22+ | 33+ | 3 | 3 | 3 | 3 | | | | 53 | 5 | S | MSS | MS |
| Marquillo | *Lr10,Lr20,N* | 2 | 3 | 3 | 3 | 22+ | 22+ | 3 | 3 | 3 | 3 | | *Lr10* | | 24 | 3 | MR | MRMS | MS |
| AC Vista | *Lr3,Lr14a,N* | 2 | 3 | 3 | 2 | X | 33+ | ;1 | 3 | 3 | 3 | | | | 29 | 5 | MR | MSS | S |
| HY358 | *Lr3,Lr14a,N* | ; | 3 | 3 | 2 | X | 33+ | x | 3 | 33+ | 3 | | | | 21 | 2 | R | MS | MS |
| Fu Mai No 3 | *Lr1,Lr15,N* | 2 | 3 | 3 | 3 | 33+ | 2 | ;1 | 3 | 23 | 3 | *Lr1* | | | 37 | 4 | MS | MS | R |
| Bajio | *Lr1,Lr14a,N* | 0 | 3 | 3 | 12 | 0 | 33+ | X | 3 | 3 | 3 | *Lr1* | | *Lr34* | 18 | 3 | MR | MS | MS |
| Victoria INTA | *Lr1,Lr14a,N* | 1 | 3 | 2 | 3 | ; | 33+ | ; | 3 | 3- | 3 | *Lr1* | | | 16 | 3 | MS | MS | MS |
| Tezanos Printos Precoz | *Lr3,Lr20,N* | 0 | 3 | 2 | 2 | 21 | 33+ | 3 | 3 | 3 | 3 | | | *Lr34* | 6 | 1 | MR | MRMS | R |
| Fortaleza | *Lr3,Lr20,N* | 2 | 3 | 2 | 1 | 0 | 33+ | 3 | 3 | 33+ | 3 | | | *Lr34* | 3 | 1 | R | R | R |
| Helvia | *Lr10,Lr14a,N* | 2 | 3 | 3 | 3 | 22+ | 22+ | ;1 | 3 | 3 | 3 | | *Lr10* | | 56 | 5 | S | MR | ? |
| Duiker | *Lr1,Lr14a,* | 2 | 2 | 3 | 3 | 22+ | 33+ | x | 3 | 3 | 3 | *Lr1* | | | 71 | 3 | MR | MRMS | MS |

| Accession name | Seedling genes | BBBD | MBDS | MGBJ | TJBJ | TDBG | MBRJ | PBDQ | THMJ | TNRJ | TCRJ | Marker data | | | MRS | SE | HR | | |
|---|---|---|---|---|---|---|---|---|---|---|---|---|---|---|---|---|---|---|---|
| | | | | | | | | | | | | *Lr1* | *Lr10* | *Lr34* | | | WPG | GLN | POR |
| | *Lr28* | | | | | | | | | | | | | | | | | | |
| Columbus | *Lr3,Lr15,N* | 1 | 3 | 3 | 3 | 33+ | 2 | ;12 | 3 | 21 | 2 | | | | 49 | 5 | MRMS | MS | S |
| Yang Mai No.1 | *Lr3,Lr15,N* | 2 | 3 | 3 | 3 | 33+ | 33+ | ;1 | 3 | 12 | 3 | | | | 46 | 5 | MS | MSS | S |
| Buck Nandu | *Lr10,Lr28,N* | 0 | 1 | 3 | 3 | 33+ | 1 | ; | 3 | 33+ | 2 | | *Lr10* | | 23 | 5 | MSS | MR | MS |
| Universal II | *Lr1,Lr28,N* | 2 | 0 | 2 | 2 | 33- | 33+ | ;2 | 3 | 3 | 3 | *Lr1* | | | 14 | 4 | S | R | MS |
| Trintecinco | *Lr10,Lr20,N* | 1 | 3 | 2 | 2 | 0 | 22+ | 3 | 3 | 33+ | 3 | | *Lr10* | | 11 | 3 | MR | MRMS | MS |
| Cinquentenario | *Lr3,Lr14a,N* | 2 | 3 | 3 | 2 | 22+ | 22+ | ;1 | 3 | 33+ | 3 | | | | 7 | 2 | MR | MS | R |
| Burnside | *Lr10,Lr3ka/30* | 1 | 1 | ; | ; | 0 | 33+ | 0 | 3 | 33+ | 3 | | *Lr10* | | 4 | 1 | MR | R | R |
| Tambillo 1 | *Lr20,Lr28,N* | 1 | 2 | 2 | ? | 21 | 2 | x | 3 | 33+ | ;12 | | | | 5 | 1 | ? | ? | R |
| Oslo | *Lr3,Lr20,N* | 1 | 3 | 1 | 2 | X | 2; | 33+ | 3 | ; | 2 | | | | 24 | 4 | MR | MS | ? |
| CN12358 | *Lr3,Lr15,N* | ; | 3 | 1 | 2 | 3 | 1 | 3 | ; | 0 | ;1 | | | | 6 | 2 | MR | R | R |
| Americano 44D | *Lr1,Lr20,N* | 3 | 3 | 2 | 2 | 12; | 2 | 33+ | 3 | 3 | 3 | *Lr1* | | | 8 | 3 | MRMS | R | R |
| Alfy 2 | *Lr1,Lr20,N* | 3 | 2 | 3 | 3 | 21 | 33+ | 33+ | 3 | 3 | 3 | *Lr1* | | | 48 | 5 | S | S | MSS |
| Wheaton | *Lr1,Lr15,N* | 3 | 3 | 1 | 3 | 33+ | 1 | ;1 | 3 | ; | 23 | *Lr1* | | | 30 | 5 | R | S | ? |
| Major | *Lr1,Lr20,N* | 3 | 2 | 3 | 3 | 2 | 33+ | 3 | 3 | 3 | 3 | *Lr1* | | | 42 | 6 | MRMS | R | R |
| Indur compactum | *Lr10,Lr20,N* | 3 | 2 | 1 | 3 | 0 | 1 | 3 | 3 | 3 | 3- | | *Lr10* | | 57 | 7 | S | S | MSS |

| Accession name | Seedling genes | BBBD | MBDS | MGBJ | TJBJ | TDBG | MBRJ | PBDQ | THMJ | TNRJ | TCRJ | Marker data | | | MRS | SE | HR | | |
|---|---|---|---|---|---|---|---|---|---|---|---|---|---|---|---|---|---|---|---|
| | | | | | | | | | | | | *Lr1* | *Lr10* | *Lr34* | | | WPG | GLN | POR |
| Rio Negro | *Lr10,Lr14a,N* | 3 | 3 | 3 | 2 | X | 33+ | ; | 3 | 3 | 3 | | *Lr10* | *Lr34* | 3 | 1 | R | R | R |
| Warigo | *Lr10,Lr20,N* | 3 | 3 | 3 | 3 | 22+ | 3 | 3 | 3 | 3 | 3 | | *Lr10* | | 44 | 5 | MS | SMS | MS |
| Cobbs 1066 | *Lr10,Lr20,N* | 3 | 3 | 3 | 3 | 22+ | 33+ | 3 | 3 | 3 | 3 | | *Lr10* | | 23 | 4 | MS | MS | MS |
| Kenya | *Lr1,Lr10,N* | 3 | 3 | 3 | 2 | 3 | 33+ | 3 | 3 | 3 | 3 | *Lr1* | *Lr10* | | 46 | 5 | MSS | S | S |
| Bencubbin | *Lr1,Lr10,Lr20* | 3 | 3 | 3 | 3 | X | 33+ | 3 | 3 | 3 | 3 | *Lr1* | *Lr10* | | 45 | 6 | S | S | MS |
| Klein Trou | *Lr1,Lr10,Lr20* | 3 | 3 | 3 | 33+ | ; | 33+ | 33+ | 3 | 3 | 3 | *Lr1* | *Lr10* | | 34 | 6 | MS | ? | MS |
| Subgroup 4 | | | | | | | | | | | | | | | | | | | |
| Frontana | *Lr10,Lr20, Lr28,N* | 1 | 2 | 3 | 2 | 22+ | 33+ | 3 | 3 | 3 | 3 | | *Lr10* | *Lr34* | 3 | 1 | R | R | R |
| CN37599 | *Lr10,Lr15, Lr20,N* | 1 | 3 | 2 | 3 | ; | 2 | 3 | 3 | ;12 | 33- | | *Lr10* | | 31 | 3 | MS | S | MS |
| Bage | *Lr1,Lr10,Lr20,N* | 2 | 3 | 2 | 2 | 0 | ;1 | 33+ | 3 | 3- | 3 | *Lr1* | *Lr10* | *Lr34* | 22 | 5 | MS | S | R |
| Katepwa | *Lr1,Lr10, Lr3ka or Lr30* | 1 | 1 | 2 | 2 | 22+ | 33+ | x | 3 | 3 | 3 | *Lr1* | *Lr10* | | 36 | 5 | MR | MS | MS |
| CN12229 | *Lr10,Lr2c, Lr15,N* | 1 | 2 | 12 | 3 | 33- | 22+ | 3 | 3 | ;12 | 21 | | | | 18 | 4 | MRMS | R | MS |
| Klein Sendero | *Lr15,Lr20,* | 1 | 2 | ; | ; | 21; | 1 | 33+ | 3 | 0 | ; | | | | 14 | 4 | MR | R | MS |

| Accession name | Seedling genes | BBBD | MBDS | MGBJ | TJBJ | TDBG | MBRJ | PBDQ | THMJ | TNRJ | TCRJ | Marker data | | | MRS | SE | HR | | |
|---|---|---|---|---|---|---|---|---|---|---|---|---|---|---|---|---|---|---|---|
| | | | | | | | | | | | | *Lr1* | *Lr10* | *Lr34* | | | WPG | GLN | POR |
| | *Lr28,N* | | | | | | | | | | | | | | | | | | |
| CN40895 | *Lr10,Lr15, Lr20,N* | 3 | 2 | ; | 1 | 22+ | 1 | 3 | ;12 | ;12 | ;12 | | *Lr10* | | 8 | 3 | ? | MR | R |
| Koalisie | *Lr1,Lr10,Lr20,N* | 3 | 2 | 3 | 3 | 22+ | 33+ | 33+ | 3 | 3 | 3 | *Lr1* | *Lr10* | | 21 | 5 | ? | MSS | S |
| Dromedaris | *Lr1,Lr10,Lr20,N* | 3 | 3 | 2 | 3 | 0 | 33+ | 3 | 3 | 3 | 3 | *Lr1* | *Lr10* | | 53 | 4 | ? | ? | MS |
| Stoa | *Lr10,Lr2c, Lr15,N* | 0 | ; | ; | 3 | 33+ | 0 | 3 | ; | ;1 | 0 | | *Lr10* | | 46 | 4 | MRMS | MS | S |
| Nordic | *Lr1,Lr10,Lr2a,Lr1 5,N* | 0 | 2 | 2 | 3 | 33- | 2 | 2 | 3 | ; | 2 | *Lr1* | *Lr10* | | 16 | 3 | MRMS | MS | R |
| NING 7840 | *Lr1,Lr14b, Lr15,Lr20,N* | 3 | 2 | ; | 12 | 22+ | ; | ; | 33+ | ; | 3 | *Lr1* | | *Lr34* | 3 | 1 | MR | R | R |

Infection types: 0 = no flecks or uredinia, 0; = faint hypersensitive flecks, ; = hypersensitive flecks, 1 = small uredinia with necrosis, , 2 = small to medium uredinia with necrosis, 3 = moderate to large size uredinia with/without chlorosis, 4 = very large uredinia without chlorosis, “+” = indicates slightly larger uredinia, “-“ = indicates slightly smaller uredinia, ITs with two symbols denote a range in IT: 22+= indicates a mixture of 2 size uredinia with chlorosis and slightly larger uredinia with chlorosis. Host response (HR): 0 = no flecks or uredinia, R = small uredinia with necrosis, MR = moderate size uredinia with necrosis, MRMS = small to medium size uredinia with moderate sporulation, MS = moderate size uredinia with chlorosis, S = large uredinia. MRS: Maximun rust severity. SE: standard error. ?: missing data

**Table S4.** Analysis of variance of rust severity rating of 275 accessions of a world collection of wheat tested in nine environments

| Source of Variation | DF | SS | MS | F Value | Pr>F | Variance components | % |
|---|---|---|---|---|---|---|---|
| Envivronments | 8 | 231276 | 28909.0 | 20.6 | <.0001 | 57.5 | 7.7 |
| Accessions | 274 | 1526625 | 5571.6 | 11.7 | <.0001 | 324.3 | 43.4 |
| With *Lr34* | 51 | 168505 | 3304.0 | 8.6 | <.0001 | | |
| Without *Lr34* | 222 | 1235863 | 5566.9 | 11.3 | <.0001 | | |
| With vs. without *Lr34* | 1 | 122257 | 122257 | 553 | <.0001 | | |
| Block(Environment) | 9 | 10188 | 1132.0 | 5.1 | <.0001 | 3.9 | 0.5 |
| Environments by Accessions | 2102 | 1019685 | 485.1 | 2.2 | <.0001 | 140.5 | 18.8 |
| Residual | 2105 | 465480 | 221.1 | . | . | 221.1 | 29.6 |

S.E. Mean = 0.41
